# Controlled synthesis of highly-branched plasmonic gold nanoparticles through peptoid engineering

**DOI:** 10.1038/s41467-018-04789-2

**Published:** 2018-06-13

**Authors:** Feng Yan, Lili Liu, Tiffany R. Walsh, Yu Gong, Patrick Z. El-Khoury, Yanyan Zhang, Zihua Zhu, James J. De Yoreo, Mark H. Engelhard, Xin Zhang, Chun-Long Chen

**Affiliations:** 1 Physical Sciences Division, Pacific Northwest National Laboratory, Richland, WA 99352 USA; 2 College of Chemistry & Chemical Engineering, Linyi University, Linyi, Shandong 276005 China; 3 Department of Mechanical Engineering, Texas Tech University, Lubbock, TX 79409 USA; 4 Institute for Frontier Materials, Deakin University, Geelong, VIC 3216 Australia; 5 Environmental Molecular Sciences Laboratory, Pacific Northwest National Laboratory, Richland, WA 99352 USA; 6 Departments of Materials Science and Engineering and of Chemistry, University of Washington, Seattle, WA 98195 USA

## Abstract

In nature, specific biomolecules interacting with mineral precursors are responsible for the precise production of nanostructured inorganic materials that exhibit complex morphologies and superior performance. Despite advances in developing biomimetic approaches, the design rules for creating sequence-defined molecules that lead to the synthesis of inorganic nanomaterials with predictable complex morphologies are unknown. Herein we report the design of sequence-defined peptoids for controlled synthesis of highly branched plasmonic gold particles. By engineering peptoid sequences and investigating the resulting particle formation mechanisms, we develop a rule of thumb for designing peptoids that predictively enabled the morphological evolution from spherical to coral-shaped nanoparticles. Through a combination of hyperspectral UV-Vis extinction microscopy and three-photon photoemission electron microscopy, we demonstrate that the individual coral-shaped gold nanoparticles exhibit a plasmonic enhancement as high as 10^5^-fold. This research significantly advances our ultimate vision of predictive bio-inspired materials synthesis using sequence-defined synthetic molecules that mimic proteins and peptides.

## Abstract (web summary)

Peptoids are promising crystallization agents, as they offer the molecular recognition capabilities of proteins and peptides but with higher stability and synthetic tunability. Here, the authors show that sequence-defined peptoids can controllably template the formation and shape evolution of gold nanostructures with defined morphologies.

## Introduction

Natural organisms produce a wide variety of exquisitely complex, nano-, micro-, and macroscale functional materials at high yields in an energy-efficient and highly reproducible manner, all under rather mild aqueous synthetic conditions^1–3^. Throughout these processes, specialized proteins and peptides are responsible for precisely controlling crystal nucleation, growth kinetics, phase and morphology, ultimately giving rise to biominerals with versatile functions. Inspired by nature, many protein- and peptide-based synthetic methods have been developed for the preparation of nanostructured inorganic materials^1,2,4,5^. These approaches are attractive because they generate complex, functional nanomaterials under mild synthetic conditions. The unique integration of bio- and inorganic nanomaterials has demonstrated superior performance^1,2,6^. Despite these advances, the rules governing design of proteins and peptides that yield nanomaterials with well-defined structures remain elusive. To some extent, this is because the complex folding of proteins and peptides make the prediction of their functions rather difficult^1,2,7^. Furthermore, although proteins and peptides can provide improved solubility, biocompatibility, and bio-targeting to inorganic nanomaterials, they are often problematic for applications due to their poor thermal and chemical stabilities^8–11^. To overcome these challenges, we will need biomimetic systems that can confer a similar level of molecular recognition, possess a higher stability, while also featuring greater simplicity in terms of tuning their functions.

Peptoids, namely poly-N-substituted glycines^12^, are good candidates in this regard: they are sequence-defined, biocompatible, and highly stable^12,13^. They can be cheaply and efficiently synthesized through a submonomer synthetic method, and several hundred commercially available amines can be used to attain large side-chain diversity^12,14^. Libraries of peptoids have been demonstrated to be rich sources of protein-binding ligands^15^ and are non-immunogenic in mice^13^. While exhibiting peptide- and protein-like molecular recognition^12,15,16^, peptoids offer unique advantages for controlling crystallization because the lack of backbone hydrogen bonding allows the explicit introduction of interactions through the side chains, thereby leading to functions with high predictability^17–21^.

**Fig. 1:**
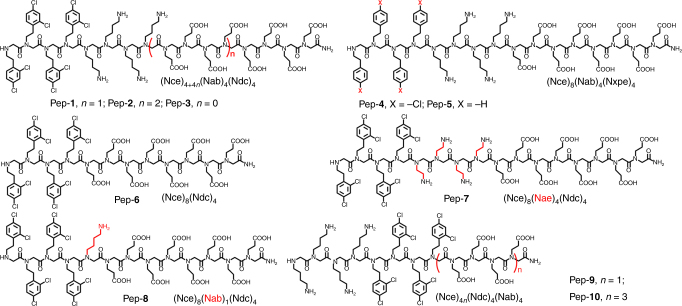
Structures of peptoids Pep-1–Pep-10. Nce = N-(2-carboxyethl)glycine, Nab = N-(4-aminobutyl)glycine, N_X_pe = N-[2-(4-X-phenyl)ethyl]glycines, Ndc = N-[2-(2,4-dichlorophenyl)ethyl]glycines, and Nae = N-(4-aminoethyl)glycine

Herein we report the design of peptoids that lead to the controlled synthesis of highly branched plasmonic gold nanoparticles. By systematically varying the hydrophobicity, number of carboxylate and amino groups, and side-chain positions of peptoids (Fig. 1), and investigating the particle formation mechanisms using in situ transmission electron microscopy (TEM), molecular dynamics simulations and time-of-flight secondary ion mass spectrometry (ToF-SIMS) techniques, we developed a rule of thumb for designing peptoids that predictively enabled the morphological evolution from spherical to coral-shaped nanoparticles. Through a combination of hyperspectral UV-Vis extinction microscopy and three-photon photoemission electron microscopy (TP-PEEM), we demonstrate that individual spherical coral-shaped gold nanoparticles exhibit a plasmonic enhancement as high as 10^5^-fold. We further highlighted the broad utility of this peptoid-based approach to enable the controlled synthesis of spherical coral-shaped Pd- and Pt- nanoparticles.

## Results

### Peptoid-induced formation of highly branched gold nanoparticles

We decided to first design peptoids for control over the synthesis of gold nanomaterials because they are model systems for understanding bio-inspired crystallization mechanisms^22,23^. Moreover, they are biocompatible and exhibit unique optical and electronic properties (e.g., plasmonics) that can be used for chemical and biological imaging applications^24,25^. In the peptoid design reported herein, we exploited the hydrophilic monomer Nce to mimic aspartic or glutamic amino acids, Nxpe to mimic hydrophobic amino acids (e.g., phenylalanine), and Nab or Nae to mimic lysine residues (Fig. 1). Ten peptoids were synthesized with the variations of (hydrophilic) carboxyl [Nce = N-(2-carboxyethl)glycine] and amino [Nab = N-(4-aminobutyl)glycine or Nae = N-(4-aminoethyl)glycine), and (hydrophobic) aromatic Nxpe side chains [N_X_pe = N-[2-(4-X-phenyl)ethyl]glycines] (Fig. 1, see Supplementary Methods for details).

**Fig. 2:**
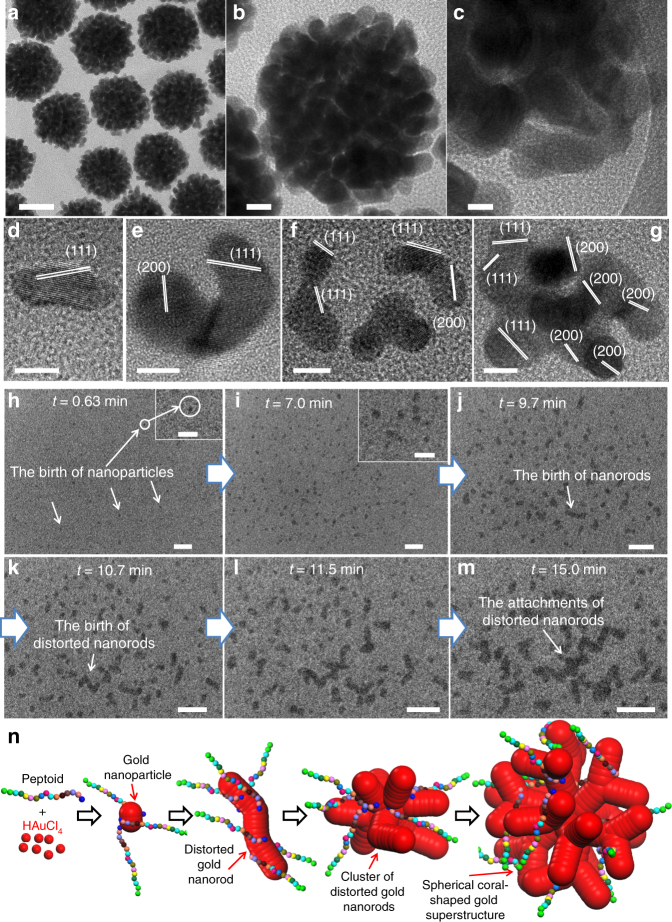
Pep-1-induced formation of spherical coral-shaped gold nanoparticles. **a**, **b** TEM images with different magnifications showing monodispersed spherical coral-shaped nanoparticles (scale bar, 50 nm for **a**, 10 nm for **b**). **c** HR-TEM revealing that the coral-shaped particles are composed of distorted nanorods (scale bar, 5.0 nm). **d**–**g**, Four high-resolution TEM images of distorted gold nanorods and clusters of nanorods formed in early stages of spherical coral-shaped particle formation, in which nanorods with (111) lattice fringes are presented (**d**) and clusters of nanorods exhibit both (111) and (200) fringes (**e**–**g**) (scale bar, 5.0 nm). **h**–**m** In situ liquid cell TEM time series (scale bar, 20 nm) showing the birth of gold NPs (**h**, **i**), nanorods (**j**), distorted nanorods (**k**), and the clusters of distorted nanorods (**l**, **m**) during early stages of coral-shaped particle formation; top right corner insets of **h**, **i** are the high-magnification TEM images showing the gold NPs (scale bar, 10 nm). **n** The proposed model showing Pep-**1**-induced spherical coral-shaped nanoparticle formation via the random attachment of distorted nanorods

To synthesize gold nanomaterials, Pep-**1** was first dissolved in HEPES buffer [pH = 7.3 ± 0.1; HEPES = 4-(2-hydroxyethyl)-1-piperazineethanesulfonic acid], and hydrogen tetrachloroaurate-(III) (HAuCl_4_) was added to initiate nanoparticle (NP) formation. HEPES was used because it is a weak capping and mild reducing reagent^26^, and thus the contributions of peptoids were significant enough to observe. TEM data showed that Pep-**1** induced the formation of monodisperse spherical coral-shaped nanoparticles (Fig. 2a; average particle diameter of 87.9 ± 14.7 nm: Supplementary Fig. 1) composed of distorted nanorods (Fig. 2b; average nanorod diameter of 7.5 ± 1.1 nm: Supplementary Fig. 2). High-resolution TEM (HR-TEM) studies further confirmed this structure (Fig. 2c) and revealed the distinct contrast between its core and peripheral branches. In the absence of Pep-**1**, the same reaction conditions led to the formation of irregular NPs (Supplementary Fig. 3), demonstrating the significance of Pep-**1** in the hedgehog particle formation. TEM results of early stages showed that Pep-**1**-induced formation of distorted gold nanorods with (111) lattice fringes (Fig. 2d) and clusters of nanorods with both (111) and (200) lattice fringes (Fig. 2e–g, Supplementary Fig. 4), indicating that distorted nanorods were randomly attached together (non-oriented attachment)^27^ to form clusters. Assembly of Pep-**1** itself into a pre-organized structure that could guide nanoparticle assembly was not observed (Supplementary Fig. 5a), and similar coral-shaped particles formed at 60 °C (Supplementary Figs. 5b, c) or under continuously stirring conditions (Supplementary Fig. 5d). These coral-shaped particles are highly stable and remained intact after being exposed to 60 °C in water for 30 h (Supplementary Fig. 5e) or in 1.0 M aqueous NaCl solution for 5 days (Supplementary Fig. 5f).

### Early stages of peptoid-induced gold nanoparticle formation

To know more about these early stages and further confirm the random attachment, we used liquid phase TEM (LP-TEM) to directly observe the particle formation in situ. Figure 2h–m shows sequential images depicting the formation of clusters of distorted nanorods (also see Supplementary Movies 1 and 2 for details), in which four distinct stages were identified. During the very early stage (*t* = 0.63 min, Fig. 2h), many small gold NPs were formed. These particles increased in number and grew with time (*t* = 7.0 min, Fig. 2i) before merging to form nanorods (*t* = 9.7 min, second stage, Fig. 2j). Further particle attachment resulted in formation of distorted nanorods (*t* = 10.5 min, third stage, Fig. 2k). Interestingly, these distorted nanorods randomly attached together (*t* = 11.5 min, Fig. 2l) to form clusters (*t* = 15 min, fourth stage, Fig. 2m) similar to those in Fig. 2d–g and Supplementary Fig. 4, indicating they are intermediates of coral-shaped particles. Unfortunately, direct observation of growth of these intermediates into the final spherical coral-shaped morphology using LP-TEM was unsuccessful, because, as they grew, the clusters fell out of the focal plane.

Based on these results, we hypothesize that random attachment of nanorods is due to Pep-**1** binding onto the nanorod surfaces, and Pep-**1** hydrophobicity and gold binding affinity are critical for nanorod formation and attachment during spherical coral-shaped particle formation (Fig. 2n).

### Engineering peptoid sequences for morphology control

To test the roles of Pep-**1** hydrophobicity and binding affinity in the formation of spherical coral-shaped gold superstructures, we synthesized two peptoids by varying the number Nce groups (Nce)_*n*_(Nab)_4_(Ndc)_4_ (Pep-**2**, *n* = 12; Pep-**3**, *n* = 4) while maintaining the same hydrophobic domains. Because they have similar hydrophobicity (Supplementary Fig. 6) and the Nce group is a weak binder to the gold surface^22^, we reasoned that such variation would not disrupt the formation of coral-shaped nanoparticles. As expected, Pep-**2** and Pep-**3** both induced the formation of coral-shaped superstructures (Supplementary Fig. 7). Because Pep-**1**–Pep-**3** all have the same hydrophobic domains (Ndc)_4_, they are expected to exhibit similar hydrophobic interactions with gold nanomaterial surfaces. At pH 7.3, all the carboxyl groups are deprotonated and interact with gold surfaces through electrostatic interactions^17^. While the influence of electrostatic interactions as a result of varying the number of carboxyl groups is minor for nanorod attachment, we believe the presence of hydrophobic interactions of (Ndc)_4_ is critical for the nanorod attachment, consistent with our hypothesis illustrated in Fig. 2n.

**Fig. 3:**
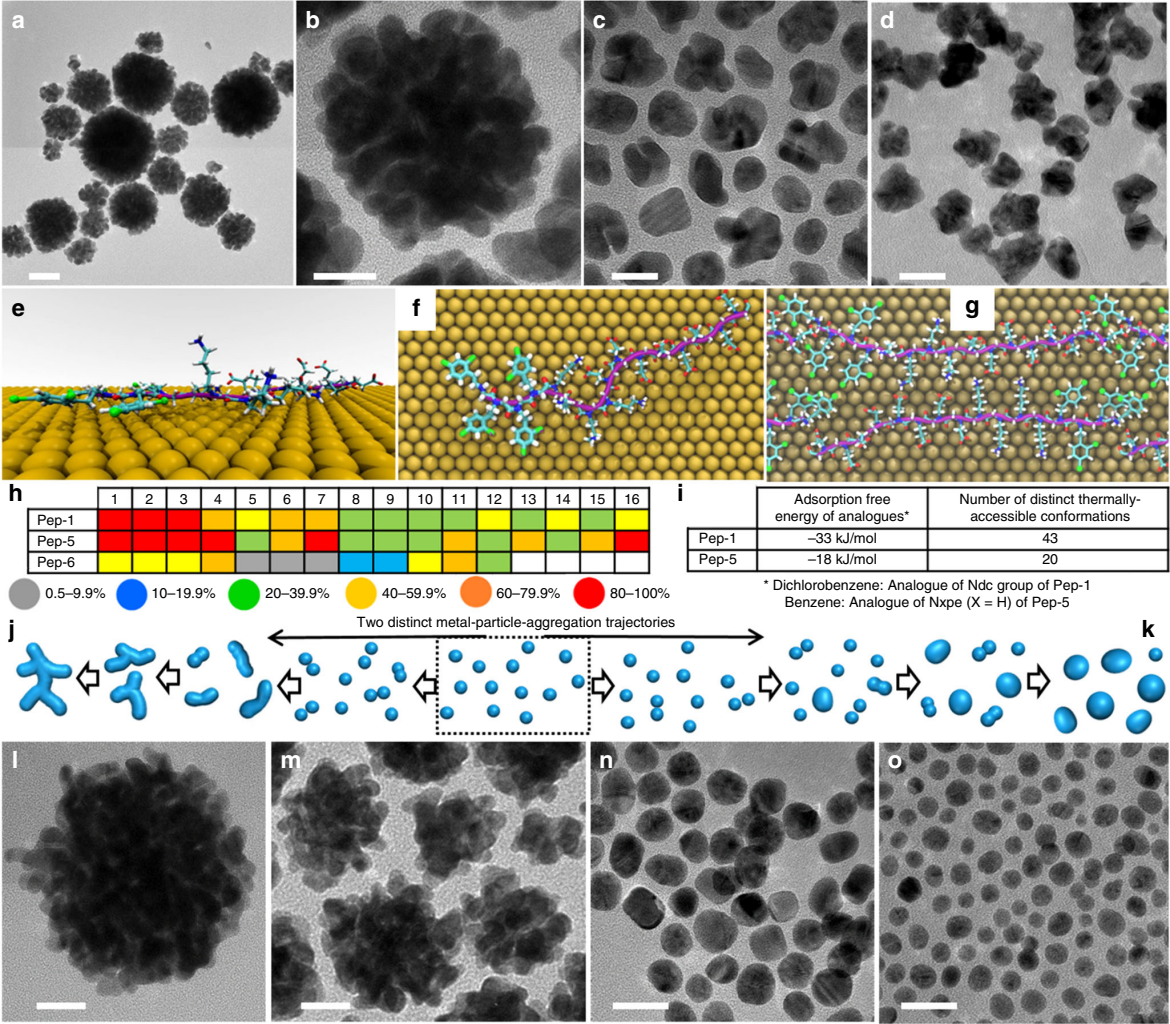
Predictable morphological evolution of gold nanomaterials induced by peptoids. **a**–**d** TEM images (scale bar, 50 nm for a, and 20 nm for **b**–**d**) of gold nanomaterials induced by Pep-**4** (**a**, **b**), Pep-**5** (**c**), and Pep-**6** (**d**). **e**–**g** Representative structures of Pep-**1** adsorbed at the aqueous Au(111) interface predicted from REST-MD simulations in which (**e**, **f**) are single chain, side and plan views and (**g**) is two-chain plan view indicating favorable inter-peptoid contacts. Color code: C, cyan; O, red; N, blue; Cl, green; the peptoid backbone is colored purple. Water molecules are not shown for clarity. **h** Average degree of peptoid-surface contact, on a residue-by-residue basis, predicted by REST-MD simulations for each of Pep-**1**, Pep-**5**, and Pep-**6** adsorbed at the aqueous Au(111) interface. The side-chain residue numbers were enumerated from the N- to C-termini. **i** The adsorption free energy of both dichlorobenzene and benzene, as analogues of Ndc of Pep-**1** and Nxpe (X = H) of Pep-**5** respectively, at the aqueous Au(111) interface; The number of distinct thermally accessible conformations of Pep-**1** and Pep-**5** generated from a clustering analysis of surface-adsorbed Pep-**1** and Pep-**5** (Supplementary Methods and Supplementary Table 1). **j**, **k** The schemes showing two distinct, well-known metal-particle-aggregation trajectories, in which the slow aggregation and fast relaxation of metal NPs lead to the fusion and formation of dumbbell structures that then relax back to spherical shapes (**k**)^35,36^; when aggregation is rapid, but relaxation is slow, dendritic structures typical of diffusion-limited aggregation form (**j**)^37,38^. TEM images of gold nanomaterials (scale bar, 20 nm) induced by Pep-**7** (**l**), Pep-**8** (**m**), Pep-**9** (**n**), and Pep-**1** at pH 5.5 (**o**) in which the predictable evolution from gold spherical coral to spherical particles produced as peptoids move from high to low binding affinities

To further test our hypothesis, Pep-**4** and Pep-**5** with varied substituent (X) of Nxpe groups (Fig. 1) were also tested. Pep-**1**, Pep-**4**, and Pep-**5** exhibited tunable hydrophobicities in the order of Pep-**1** > Pep-**4** (X = 4-chloro) > Pep-**5** (X = 4-hydrogen) (Supplementary Fig. 8). While Pep-**4**-induced formation of polydisperse coral-shaped particles (Fig. 3a, b, Supplementary Fig. 9a) composed of nearly spherical building blocks, gold nanomaterials induced by Pep-**5** exhibited an irregular morphology containing almost no nanorods (Fig. 3c, Supplementary Fig. 9b), indicating the hydrophobicity contributed from (Ndc)_4_ groups of Pep-**1** is critical for nanorod formation and attachment during coral-shaped particle formation.

To test the significance of Nab residues in Pep-**1**, Pep-**6** (Fig. 1, a Pep-**1** analogue without Nab groups) was used as a negative control. As shown in Fig. 3d and Supplementary Fig. 9c, Pep-**6** only induced formation of irregular particles, indicating Nab residues are important for Pep-**1** binding to nanorods and their attachment during coral-shaped particle formation.

### Probing interactions between peptoids and gold

To explore the underlying interactions that drive the observed behavior, we next carried out Replica Exchange with Solute Tempering Molecular Dynamics (REST-MD) simulations^28^ to elucidate the links between peptoid sequence and binding behavior. Simulations of peptoids were done at the aqueous Au(111) interface to characterize peptoid-surface adsorption at the molecular level (Supplementary Methods). Au(111) surfaces were used because Au(111) lattice fringes were observed in the nanorod building blocks (Fig. 2d–g and Supplementary Fig. 4). Furthermore, previous studies indicate that the Au(111) interface can provide a useful approximate structural model of more complex (i.e., poly-crystalline) Au interfaces under aqueous conditions when studying peptide/Au adsorption from solution^29,30^. Thus, we reason that REST-MD simulations of peptoids at the aqueous Au(111) interface can provide useful insight into the principal contact modes and conformational entropic contributions (vide infra) of peptoids adsorbed onto Au nanorod surfaces. In these simulations, we adapted a previously established peptoid force field^20^ and predicted the peptoid-Au(111) interactions by modeling the adsorption of a single chain of each peptoid in contact with the aqueous Au(111) interface using the polarizable force-field GolP-CHARMM^31^. Snapshots of a representative structure of Pep-**1** (Fig. 3e, f) reveal the close contact between the Ndc rings and the metal surface. Analogous images for other peptoids are provided in Supplementary Fig. 10.

The average degree of surface-peptoid contact for each residue (Fig. 3h) showed that Pep-**6** exhibited the weakest binding to Au(111) surfaces, confirming the significant contribution of Nab to Pep-**1**-gold binding. Multi-chain standard MD simulations of Pep-**1** adsorbed on Au(111) (Fig. 3g) further confirmed the importance of Nab. Specifically, Pep-**1** amphiphilicity supported the substantive inter-peptoid electrostatic interactions between Nce and Nab side-chain groups.

Because both Pep-**1** and Pep-**5** exhibited almost an indistinguishable degree of contact with the Au(111) surfaces (Fig. 3h), we further used well-tempered metadynamics simulations^32^ to obtain the adsorption free energy of both dichlorobenzene (analogue of Ndc of Pep-**1**) and benzene (analogue of Nxpe of Pep-**5**) at the aqueous Au(111) interface. These simulations revealed a much stronger binding of dichlorobenzene (−33 kJ/mol; Pep-**1** analogue) in contrast to that of benzene (−18 kJ/mol; Pep-**5** analogue) (Fig. 3i). Another factor that can affect binding affinity is conformational entropic contribution^33,34^. For that, we carried out analysis of surface-adsorbed Pep-**1** and Pep-**5** using a clustering approach (Supplementary Methods) which yields the set of distinct, thermally accessible conformations and their relative populations in the ensemble (Supplementary Table 1). This analysis indicates a larger number of distinct thermally accessible conformations of Pep-**1** compared to Pep-**5** (Fig. 3i and Supplementary Table 1), suggesting the more favorable binding^33^ of Pep-**1**. Taken together, our simulation results indicate that Pep-**1** binds to aqueous Au(111) interfaces much more strongly than does Pep-**5**.

To experimentally confirm the above computational predictions, we conducted ToF-SIMS studies to investigate the binding affinities of Pep-**1**, Pep-**5**, and Pep-**6** toward Au(111) surfaces in aqueous solutions. ToF-SIMS results show that Pep-**1** exhibits the strongest binding affinity (Supplementary Fig. 11), consistent with our computational predictions.

### Predictable morphological evolution of gold nanomaterials

Recent LP-TEM studies of metal NP suspensions have shown that particle interactions lead to fusion and formation of dumbbell structures that then relax back to spherical shapes, which minimizes the interfacial free energy (Fig. 3k)^35,36^. On the other hand, numerous studies of metal thin film epitaxy^37^, as well as bulk solution studies^38^ have shown that when aggregation is rapid, but relaxation is slow, dendritic structures typical of diffusion-limited aggregation form instead. Moreover, the extended nature of these structures leads to enhanced particle capture and thus a smaller number of particles with a larger average size (Fig. 3j). Spherical coral-shaped particles present morphologies that are intermediates between these two end members. When aggregation and relaxation compete, partial relaxation leads to compact cores with dendritic arms that retain the history of nanoparticle attachment events. Therefore, it is reasonable to conclude that strong Pep-**1**-gold binding and peptoid amphiphilicity drive coral-shaped particle formation. The strong binding free energy of Pep-**1** is directly correlated with low interfacial free energy^39^ and therefore low driving force for relaxation back to spherical particles. Moreover, the strong Pep-**1**-gold binding should reduce the mobility of surface atoms^40^, thus further slowing the kinetics of relaxation, while the substantive inter-peptoid electrostatic interactions (Fig. 3g) and the amphiphilicity of Pep-**1** can be expected to lead to rapid particle aggregation.

Based on the clear trends in particle morphology from spherical coral to irregular as the binding affinity or hydrophobicity of the peptoids decreases (Fig. 3h,i, Supplementary Figs. 8, 11), combined with the well-known metal-particle aggregation trajectories (Fig. 3j, k), we deduce the following principles for peptoid design: For peptoids to induce coral-shaped nanoparticle formation they need adequate hydrophobicity and strong gold binding affinity; decreasing peptoid-gold binding affinity or providing inadequate hydrophobicity leads to a trend toward formation of spherical nanoparticles. To demonstrate these principles can be used to design peptoids that lead to predictive coral-shaped particle formation, we designed two more peptoid sequences in which (Nab)_4_ was changed to (Nae)_4_ (Pep-**7**) or (Nab)_1_ (Pep-**8**), keeping the same number of Nce and Ndc groups, and a similar pattern and hydrophobicity compared to Pep-**1**. Therefore, a similar enhanced peptoid-gold binding affinity will be expected comparing to Pep-**6** (Fig. 1, a Pep-**1** analogue without Nab). As expected, both Pep-**7** (Fig. 3l, Supplementary Figs. 12a, b) and Pep-**8** (Fig. 3m, Supplementary Figs. 12c, d) induced the formation of coral-shaped nanoparticles.

As the bio-controlled crystallization process is sequence-dependent^1,2^, the arrangement of Nce, Ndc and Nab groups in Pep-**1 -** Pep-**3** ought to be one of the key elements that determine the coral-shaped particle formation. To this end, we synthesized Pep-**9** (Nce)_4_(Ndc)_4_(Nab)_4_ in which the four hydrophobic monomers of Ndc in Pep-**3** were moved from the N-terminus to the middle positions. Compared to Pep-**3**, its analogue Pep-**9** exhibited lowered gold binding affinity (TOF-SIMS results: Supplementary Fig. 11c; Simulation results: Supplementary Figs. 10c and e, where the Pep-**9**-surface contact was similar to that of Pep-**6**, Fig. 3h, but with the Ndc/Nce blocks swapped). As expected, Pep-**9** induced the formation of monodisperse and nearly spherical NPs (Fig. 3n, Supplementary Figs. 13a, b). Similar results were observed when a Pep-**2** analogue, namely Pep-**10** (Nce)_12_(Ndc)_4_(Nab)_4_, was used (Supplementary Figs. 13c, d).

To investigate the influence of the protonation state of peptoids in spherical coral-shaped nanoparticle formation, we further tested the reaction condition at pH 5.5. According to the titration curve of Pep-**1** (Supplementary Fig. 14a), Nce groups will start to become protonated. The X-ray photoelectron spectroscopy (XPS) measurements (Supplementary Table 2) showed that the binding affinity of Pep-**1** toward Au(111) decreased as pH was lowered from pH 7.3 to pH 5.5. REST-MD simulation results indicated that a single chain of Pep-**1** at pH 5.5 (where all Nce groups were protonated) yielded a comparable degree of contact with Au(111) relative to Pep-**1** at pH 7.3 (Supplementary Fig. 10c). However, multi-chain MD simulations of Pep-**1** at pH 5.5 revealed a dramatic reduction in inter-peptoid interactions (Supplementary Fig. 10d) and a loss of the tightly packed, amphiphically driven anti-parallel chain arrangement compared with Pep-**1** at pH 7.3 (Fig. 3g). As expected, at pH 5.5, Pep-**1** induced the formation of nearly spherical nanoparticles (Fig. 3o, Supplementary Figs. 14b-d).

### Plasmonic properties of coral-shaped gold nanoparticles

**Fig. 4:**
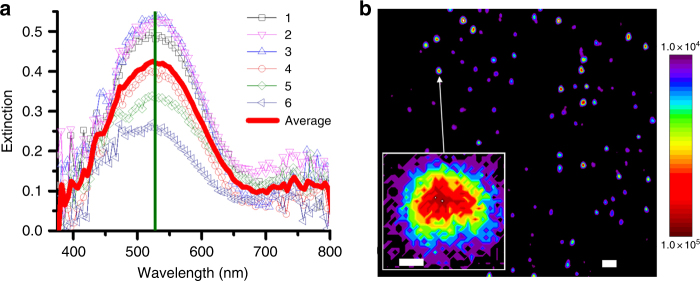
Plasmonic properties of the spherical coral-shaped gold nanoparticles induced by Pep-1. **a** Hyperspectral UV-Vis extinction of six individual particles revealing well-defined plasmon resonances centered at ~528 nm. **b** Three-photon photoemission electron micrograph of a sparse distribution of particles showing the photoemission enhancement map (scale bar, 50 nm), in which a single-coral-shaped particle (the insert) exhibited a plasmonic enhancement as high as 10^5^ (scale bar, 20 nm)

To evaluate the plasmonic properties of the Pep-**1**-induced coral-shaped gold nanoparticles, we employed a combination of hyperspectral UV-Vis extinction microscopy and TP-PEEM (Supplementary Methods). Whereas, the former reports on the plasmonic response of a single particle, the latter can be used to map the local electric fields generated from spherical coral-shaped nanoparticles with nanometer resolution. As shown in Fig. 4a and Supplementary Fig. 15a, these coral-shaped nanoparticles exhibit a well-defined plasmon resonance centered at 528 nm, indicating the uniformity of their plasmonic responses. The enhancement images (Fig. 4b, Supplementary Fig. 15b) reveal that individual coral-shaped particles support a plasmonic enhancement factor of as high as 10^5^, which is 2–3 orders of magnitudes stronger than those achieved from Pep-**5**-induced Au NPs (Supplementary Fig. 16) and other previously reported plasmonic NPs^41,42^. These data confirm that the unique plasmonic enhancement arises from the coral-shaped morphology. High-resolution TP-PEEM data (the inset of Fig. 4b, Supplementary Fig. 15b) reveal that the coral-shaped particle center comprises the most pronounced region of plasmonic enhancement, consistent with our proposed model of a spherical coral-shaped particle (Fig. 2n).

### Synthesis of other coral-shaped metallic nanoparticles

To further test the universality of our approach for controlled materials synthesis, we exploited Pep-**1** for the synthesis of Pt and Pd nanoparticles because its binding to Pt and Pd is expected to be similar to that of Au^43^. As anticipated, Pep-**1** induced the formation of both spherical coral-shaped Pt- and Pd- nanoparticles (Supplementary Fig. 17). Similarly, these Pt- and Pd- coral-shaped particles are highly stable and remained intact after being exposed to 60 °C in water for 30 h (Supplementary Fig. 18). Taken together, these results demonstrate that our approach provides a level of predictability and control in the synthesis of metallic coral-shaped particles that previously reported approaches^1–5^, including templating mechanisms, do not offer.

## Discussion

By designing sequence-defined peptoids for controlling gold nanomaterial formation and investigating their formation mechanism using in situ TEM, molecular simulations, ToF-SIMS, and peptoid engineering, we developed a rule of thumb for designing peptoids that predictively enable the morphological evolution from spherical to coral-shaped gold nanoparticles. The formation of these particles is primarily a result of using peptoids that meet three criteria: (a) possessing amino- (–NH_2_) containing side chains, (b) exhibiting strong hydrophobicity by incorporating Ndc groups, and (c) featuring the specific arrangement of Nce, Ndc, and Nab groups. During the early stages of particle formation, peptoids induce the formation of distorted nanorods and their random attachment and both peptoid-gold binding affinity and peptoid hydrophobicity are critical for morphological controls. These highly stable coral-shaped particles exhibit sharp plasmon resonances at 528 nm, and support a high-plasmonic enhancement factor on the order of 10^5^. We further highlighted the broad utility of our approach to enable the controlled synthesis of coral-shaped Pd- and Pt- nanoparticles. These findings suggest that the peptoid design principles established here could provide a basis for further design of sequence-defined molecules enabling the predictable synthesis of inorganic nanomaterials. Because peptoids are biocompatible, highly stable, and offer peptide- and protein-like molecular recognition^12^, peptoid-based approaches offer a unique platform for biomimetic synthesis of functional nanomaterials and for understanding bio-controlled crystallization.

## Methods

### Peptoid synthesis

Detailed information on materials and methods is available in Supplementary Methods. Peptoids were synthesized using a solid-phase submonomer synthesis method. They were synthesized on a commercial Aapptec Apex 396 robotic synthesizer. Peptoids were cleaved from the resin by addition of 95% trifluoroacetic acid (TFA) in water, and then dissolved in water and acetonitrile (v/v = 1:1) for HPLC purification. Lyophilized and HPLC-grade peptoids (3.0 × 10^-6^ mol) were mixed with 1.5 mL ultrapure water in a glass vial, and 10 µl saturated (NH_4_)_2_CO_3_ solutions were used to facilitate dissolution. The final concentration of peptoid stock solution was 2.0 mM.

### Preparation of gold nanomaterials

A total of 75 µl of 2 mM peptoid stock solution and 250 µl of 0.2 M HEPES solution were added into 175 µl of water. Twenty minutes later, 6 µl of HAuCl_4_ (0.1 M) was added and vortexed for 15 s, and then undisturbed at room temperature. The resulting gold nanomaterials were formed after 5 h of incubation.

### Molecular dynamics simulations

To predict the conformational ensemble of the surface-adsorbed peptoids, we performed REST-MD simulations comprising a single chain of each of three peptoids, Pep-**1** (with two different protonation states, corresponding with pH 5.5 and pH 7.3), Pep-**5**, Pep-**6**, and Pep-**9**, adsorbed at the aqueous Au(111) interface. To probe inter-chain interaction effects, we also carried out standard MD simulations of a two-chain surface-adsorbed peptoid system. Finally, to estimate and compare the adsorption free energy of dichlorobenzene and benzene, we performed multiple walker well-tempered metadynamics simulations, comprising a single adsorbate (dichlorobenzene and benzene) and the aqueous Au(111) interface. Simulation details are provided in Supplementary Methods.

### Data availability

The data that support the findings of this study are available from the corresponding author upon request.

## Electronic supplementary material

Supplementary Information

Peer Review File

Description of Additional Supplementary Files

Suplementary Movie 1

Supplementary Movie 2
