# Supplementary material for: Controlled synthesis of highly-branched plasmonic gold nanoparticles through peptoid engineering
Source: Nat Commun. 2018 Jun 13;9:2327. doi: 10.1038/s41467-018-04789-2 (PMC5998043; doi:10.1038/s41467-018-04789-2)
Supplement: Supplementary file 1 — Supplementary Information [file 41467_2018_4789_MOESM1_ESM.pdf]

Controlled synthesis of highly-branched plasmonic gold nanoparticles through peptoid engineering

Feng Yan[1, 2], Lili Liu[1, 3], Tiffany R. Walsh[4], Yu Gong[1], Patrick Z. El-Khoury[1], Yanyan Zhang[5], Zihua Zhu[5], James J. DeYoreo[1,6], Mark H. Engelhard[5], Xin Zhang[1], Chun-Long Chen[1,*].

[1] Physical Sciences Division, Pacific Northwest National Laboratory, Richland, WA 99352, USA
[2] College of Chemistry & Chemical Engineering, Linyi University, Linyi, Shandong 276005, China
[3]Department of Mechanical Engineering, Texas Tech University, Lubbock, TX 79409, USA
[4] Institute for Frontier Materials, Deakin University, Geelong, VIC 3216, Australia
[5] Environmental Molecular Sciences Laboratory, Pacific Northwest National Laboratory, Richland, WA 99352, USA
[6] Departments of Materials Science and Engineering and of Chemistry, University of Washington, Seattle, WA 98195, USA.

Correspondence to: Chunlong.Chen@pnnl.gov.

This PDF file includes:

Supplementary Methods
Supplementary Tables 1 to 2
Supplementary Figures 1 to 18

Other Supplementary Materials for this manuscript include the following:

Supplementary Movies 1 to 2

Supplementary Methods

Materials. All solvents and chemicals were of analytical grade and were obtained from commercial sources and used without further purification. 0.2M HEPES buffer (4-(2-hydroxyethyl)-1-piperazineethanesulfonic acid) was made by directly diluting 1.0 M HEPES buffer (pH = 7.3 ± 0.1; Fisher Scientific) with ultrapure water. The ultrapure water was obtained from a Milli-Q water purification system (Millipore Corp., Bedford, MA) with resistivity of 18.2 MΩ·cm.

Transmission electron microscopy (TEM) characterization. TEM images were taken on the FEI Tecnai G2 transmission electron microscope and JEM 2100 (JEOL) with an accelerating voltage of 200 kV. The samples were prepared by pipetting one drop of solution onto a 3-mm-diameter copper grid coated with carbon film.

Liquid phase TEM (LP-TEM). LP-TEM was done by using a standard Hummingbird static cell on a Hummingbird Scientific's Liquid flow holder. The liquid layer for the in situ TEM experiment was formed in the fluid stage tip by sealing two silicon chips (Hummingbird Scientific, USA), with a 50 x 200 μm^2 opening etched from the center, which had a 50 nm thick amorphous SiN membrane to form the electron transparent window for observation. The two chips are separated by a 100 nm spacer that allows the passage of incident electrons. The silicon chips were treated for 40 seconds with plasma (Harrick Plasma) cleaning to make them totally hydrophilic before assembling liquid cell. The bottom chip was firstly placed with membrane side up inside the well-cleaned fluid stage tip, and a 0.6 µL stock solution was placed onto its central spot with a pipette. The top chip was then placed membrane side down to align well with the bottom chip, and a transparent window can be observed under a stereo microscope. Finally the liquid cell holder was placed in a pump station (Pfeiffer vacuum) to test the vacuum condition before loading it to the microscope, which was kept for at least 5 minutes when the vacuum reached very well. Bright field images were recorded with an Eagle CCD detector, which was made into a movie using the movie maker software.

Time-of-flight secondary ion mass spectrometry (ToF-SIMS). ToF-SIMS measurements were acquired using a ToF.SIMS5 instrument (IONTOF GmbH, Münster, Germany) (located at PNNL, Richland, WA). For peptoid adsorption studies, freshly-cleaned Au(111) substrates (100 nm Au deposited on silicon substrates with 20 nm Cr as an adhesion layer) were incubated in corresponding peptoid aqueous solutions (pH = 7.3) for overnight, then washed thoroughly with water and dried by N_2 blowing for ToF-SIMS studies.

X-ray photoelectron spectroscopy (XPS). The peptoid-Au(111) samples used for XPS measurements were prepared using a same method that was described above to prepare TOF-SIMS samples: freshly-cleaned Au(111) substrates were incubated with corresponding peptoids aqueous solutions (pH7.3 or pH5.5) for overnight, they were then washed thoroughly with water and dried by N2 blowing for XPS studies. XPS data were acquired using a Physical Electronics Quantera Scanning X-ray Microprobe. This system uses a focused monochromatic Al Kα X-ray (1486.7 eV) source for excitation and a spherical section analyzer. The instrument has a 32 element multichannel detection system. The X-ray beam is incident normal to the sample and the photoelectron detector is at 45° off-normal. High energy resolution spectra were collected using a pass-energy of 69.0 eV with a step size of 0.125 eV. For the Ag $3d_{5/2}$ line, these conditions

produced a FWHM of 0.92 eV ± 0.05 eV. The binding energy (BE) scale is calibrated using ISO 15472 Ed. 2 Surface Chemical Analysis - XPS – Calibration of energy scales. The Cu $2p_{3/2}$ feature is set at 932.62 ± 0.05 eV and Au $4f_{7/2}$ line is set at 83.96 ± 0.05 eV. Quantification was performed using Ulvac-phi Inc., MultiPak software version 9.1.1.7

Three-photon photoemission electron microscopy (TP-PEEM). Photoemission from the spherical coral-shaped gold nanoparticles was imaged using a photoemission electron microscope (Elmitec, PEEM III). The sample is mounted approximately 2 mm from an electrically grounded objective lens. A −20 kV electronic potential is applied to the sample in order to accelerate and transfer the photoelectrons to an imaging column containing a series of electro-magnetic lenses which focus and project the photoelectron image onto a microchannel plate/phosphor screen detector. Images are acquired with a computer controlled charge-coupled device camera. The base pressure of the microscope chamber is ~9×10^{-11} Torr, and increases to ~ 5×10^{-10} Torr throughout the measurements. The sample was irradiated with laser pulses centered at 780 nm, from a 90 MHz Titanium-Sapphire femtosecond oscillator (Griffin-10, KM Labs). Following external prism pair compression transform limited pulses, of 15 fs duration, were delivered to the sample chamber. The laser is focused to a ~ 8×10^{-3} mm^2 area, and its with polarization is controlled using a half wave plate.

Hyperspectral UV-Vis extinction microscopy. This measurement was achieved by coupling a hyperspectral detector (Surface Optics Corp.) to a conventional optical microscope. In this study, the incident broad band depolarized light source was transmitted through the sample, consisting of a sparse distribution of spherical coral-shaped gold nanoparticles on a glass coverslip, using a bright field condenser. Hyperspectral extinction images are represented as $-\log(I_{\text{sample}}/I_{\text{substrate}})$, in which I_{sample} is the spatially (130 nm^2/pixel) and spectrally (375-800 nm, $\Delta\lambda$ = 4.7 nm) resolved intensity across the sample, and $I_{\text{substrate}}$ is the reference hyperspectral intensity image collected from the blank underlying glass coverslip.

Peptoid Synthesis
All peptoids were synthesized on a commercial Aapptec Apex 396 robotic synthesizer on using a solid-phase submonomer cycle as described previously.(*1, 2*) All amine submonomers and other reagents used for our peptoids synthesis are obtained from commercial sources and used without further purification. Rink amide resin (0.52 mmol/g, AappTec) was used to generate C-terminal amide peptoids. In this method, the Fmoc group on the resin was deprotected by adding 2 mL of 20% (*v*/*v*) 4-Methylpiperidine/N,N-dimethylformamide (DMF), agitating for 20 min, draining, and washing with DMF. All DMF washes consisted of the addition of 1.5 mL of DMF, followed by agitation for 1 min (repeated five times). An acylation reaction was then performed on the amino resin by the addition of 1.6 mL of 0.6 M bromoacetic acid in DMF, followed by 0.35 mL of 50% (*v*/*v*) N,N-diisopropylcarbodiimide (DIC)/DMF. The mixture was agitated for 30 min at room temperature, drained, and washed with DMF. Nucleophilic displacement of the bromide with various primary amines occurred by a 1.6 mL addition of the primary amine monomer as a 0.6 M solution in N-methyl-2-pyrrolidone (NMP), followed by agitation for 60 min at room temperature. The monomer solution was drained from the resin, and the resin was washed with DMF as described above. The acylation and displacement steps were repeated until a polypeptoid of the desired length was synthesized. All reactions were performed at room temperature. Peptoid chains were cleaved from the resin by addition of 2.0 mL 95% (*v*/*v*)

trifluoroacetic acid (TFA) in water for 35 min, which was then evaporated off under a stream of nitrogen gas. Following cleavage, peptoids were dissolved in 4.0 mL mixture (*v*/*v* = 1:1) of water and acetonitrile for further purification.

All peptoids were purified by reverse-phase HPLC on a XBridge™ Prep C18 10 μm OBD™ (10 μm, 19 mm × 100 mm), using a gradient of 5-95% acetonitrile in H_2O with 0.5% TFA over 15 min. The final products were analyzed using Waters ACQUITY reverse-phase UPLC (5–95% CH_3CN in H_2O at 0.4 mL/min over 5 min at 40°C with a ACQUITY®BEH C18, 1.7 μm, 2.1 mm × 50 mm column) that was connected with a Waters SQD2 mass spectrometry system. The final peptoid products were lyophilized at least twice from their solution in mixture (v/v = 1:1) of water and acetonitrile. All lyophilized peptoids were finally divided into small portions (3.0×10^{-6} mol) and stored at -80°C.

Information of Peptoid Sequences
Structures of the synthesized peptoids and molecular weight of each peptoid as determined by mass spectrometry are shown below. The following monomer abbreviations were used to name the sequences:

Nce: N-(2-carboxyethyl)glycine; **Ndc**: N-[2-(2, 4-dichloro phenethyl)]glycine;
Nmc: N-[2-(4-chloro phenethyl)]glycine; **Npe**: N-[2-(X-phenethyl)]glycine;
Nab: N-(4-aminobutyl)glycine; **Nae**: N-(4-aminoethyl)glycine.

Peptoid-**1** [$(Nce)_8(Nab)_4(Ndc)_4$]: 2483.0 (Molecular weight), 1242.8 (Found:$[M/2+H]^+$).

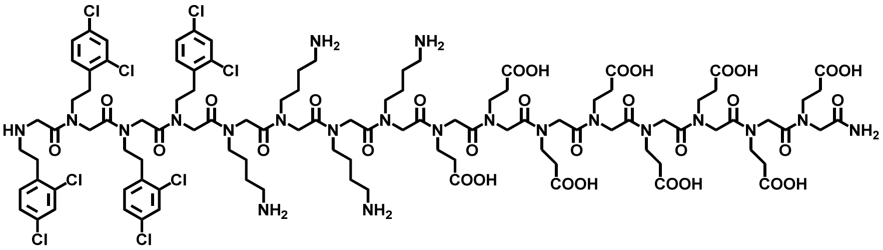

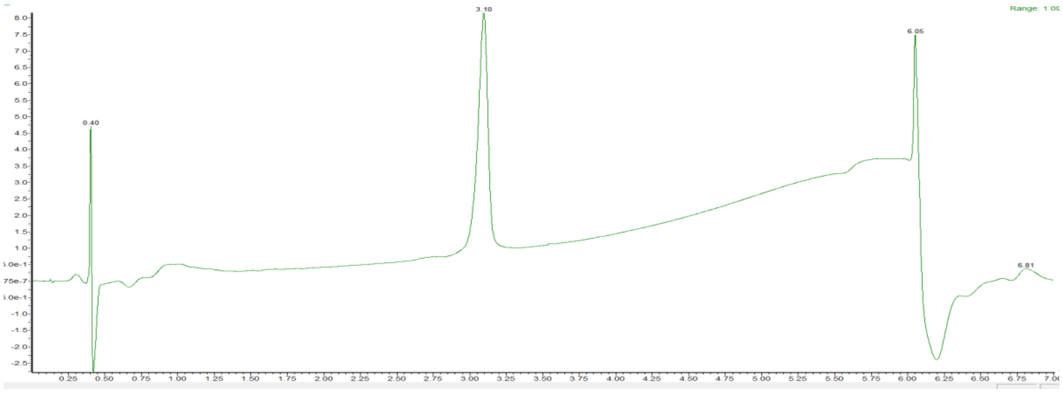

UPLC characterization of Pep-**1**

Peptoid-**2** [$(Nce)_{12}(Nab)_4(Ndc)_4$]: 2999.5 (Molecular weight), 1500.4 (Found:$[M/2+H]^+$).

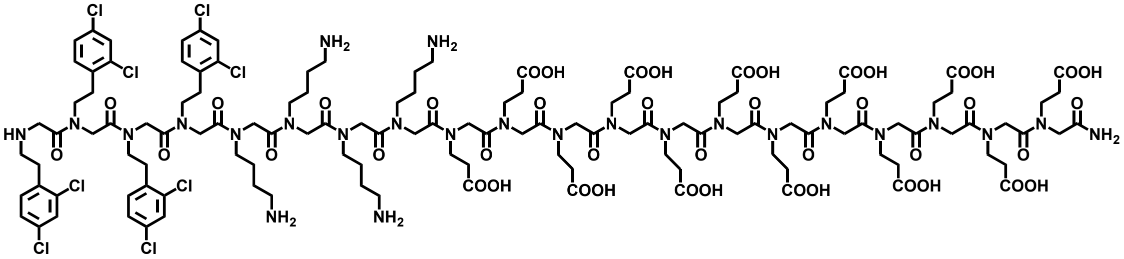

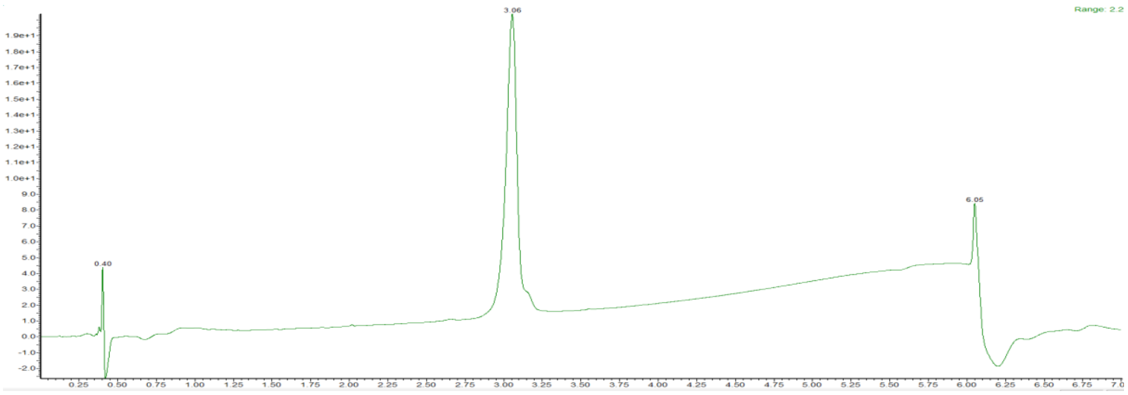

UPLC characterization of Pep-**2**

Peptoid-**3** [(Nce)$_4$(Nab)$_4$(Ndc)$_4$]: 1966.5 (Molecular weight), 993.8 (Found:$[M/2+H]^+$).

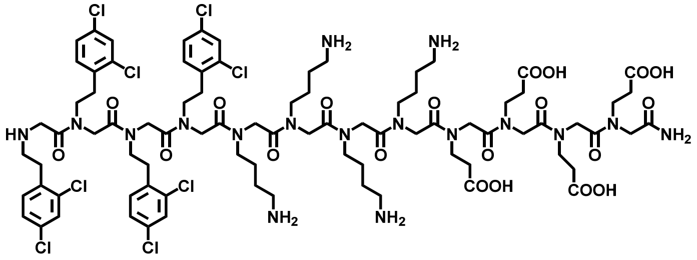

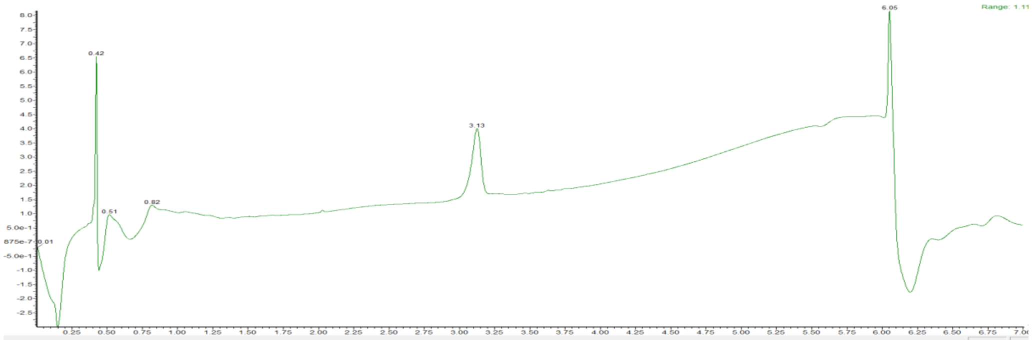

UPLC characterization of Pep-**3**

Peptoid-**4** [(Nce)$_8$(Nab)$_4$(Nmc)$_4$]: 2341.9 (Molecular weight), 1172.8 (Found:$[M/2+H]^+$).

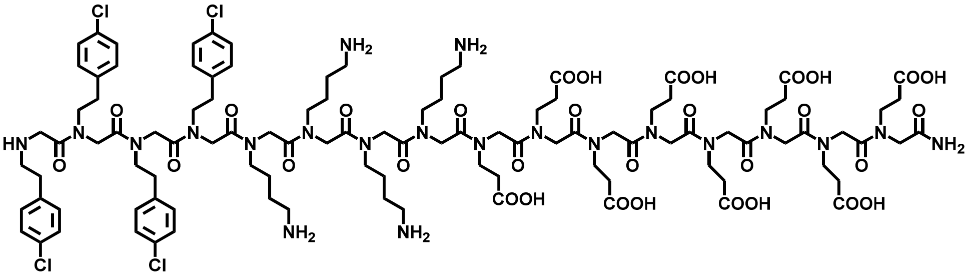

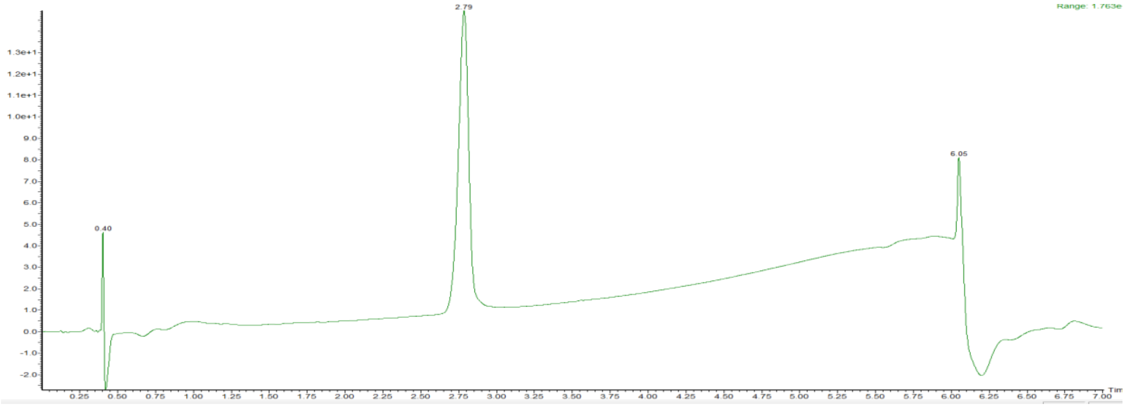

UPLC characterization of Pep-**4**

Peptoid-**5** [$(Nce)_8(Nab)_4(Npe)_4$]: 2207.5 (Molecular weight), 1104.4 (Found:$[M/2+H]^+$).

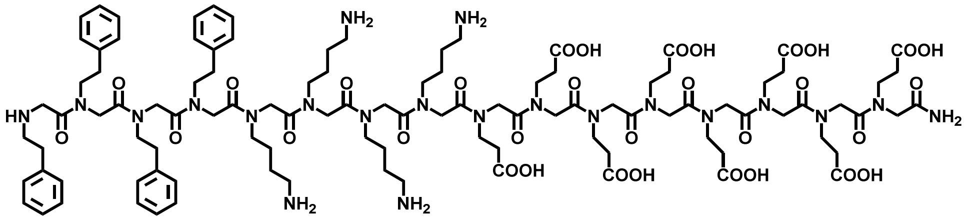

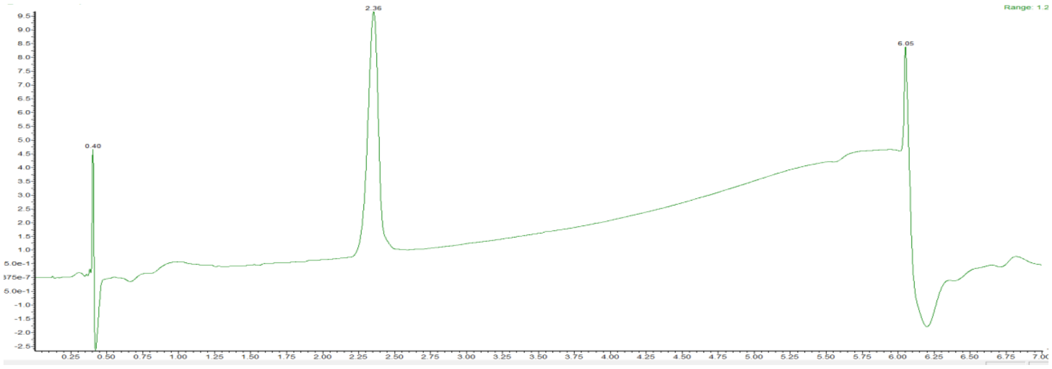

UPLC characterization of Pep-**5**

Peptoid-**6** [$(Nce)_8$ $(Ndc)_4$]: 1970.3 (Molecular weight), 1970.1 (Found:[M]).

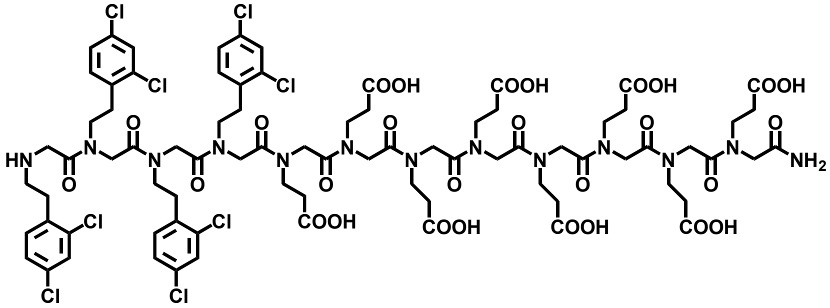

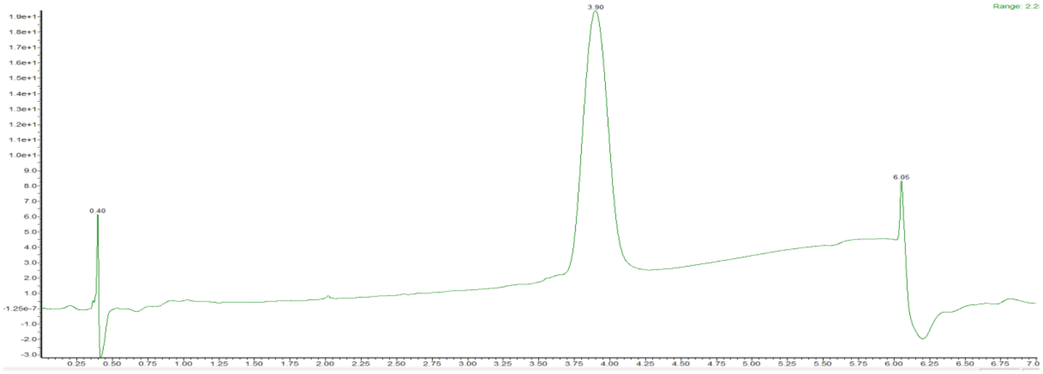

UPLC characterization of Pep-**6**

Peptoid-**7** [(Nce)$_8$ (Nae)$_4$(Ndc)$_4$]: 2370.8 (Molecular weight), 1186.1 (Found:$[M/2+H]^+$).

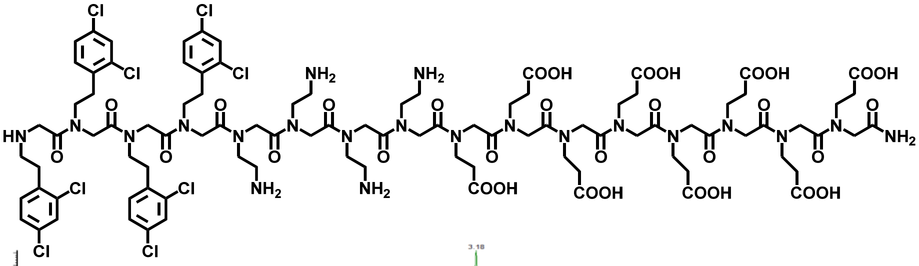

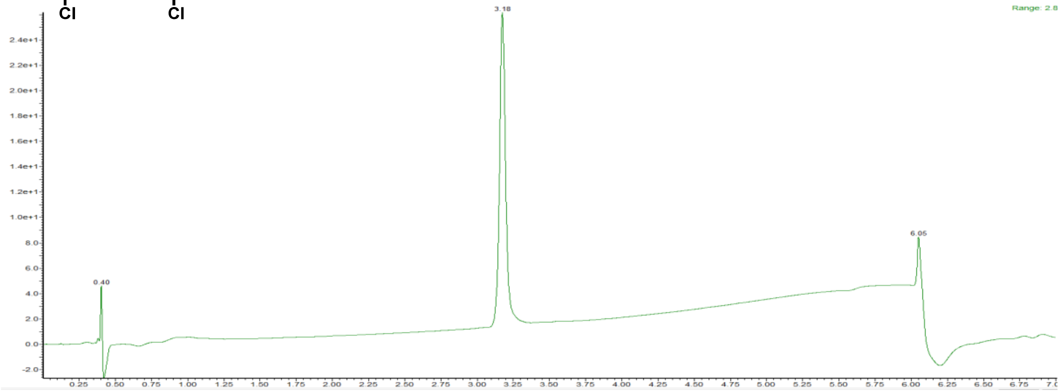

UPLC characterization of Pep-**7**

Peptoid-**8** [(Nce)$_8$ (Nab)$_1$(Ndc)$_4$]: 2098.48 (Molecular weight), 1050.05 (Found:$[M/2+H]^+$).

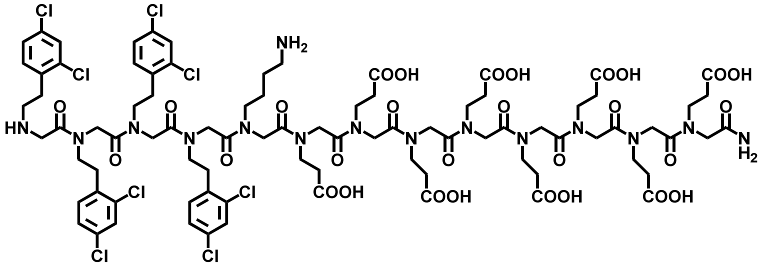

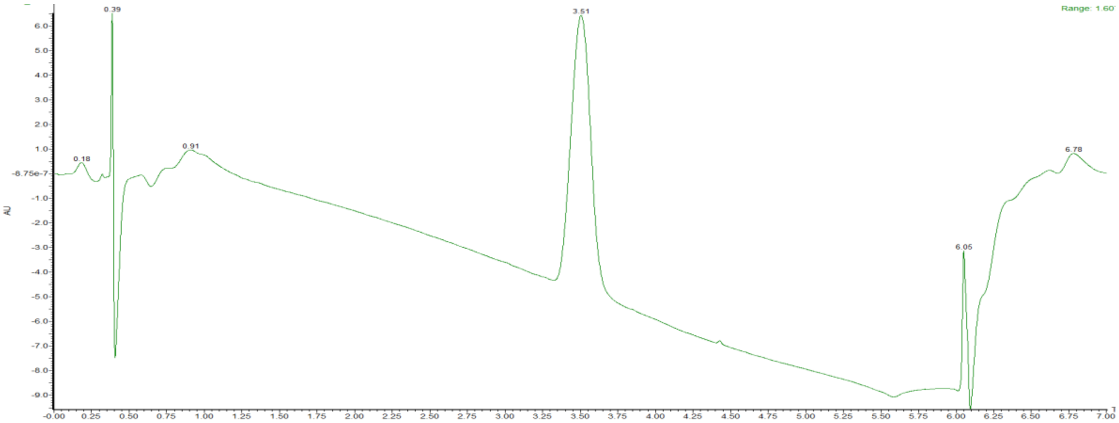

UPLC characterization of Pep-**8**

Peptoid-**9** [(Nce)$_4$ (Ndc)$_4$(Nba)$_4$]: 1966.5 (Molecular weight), 983.2 (Found:[M/2+H]$^+$).

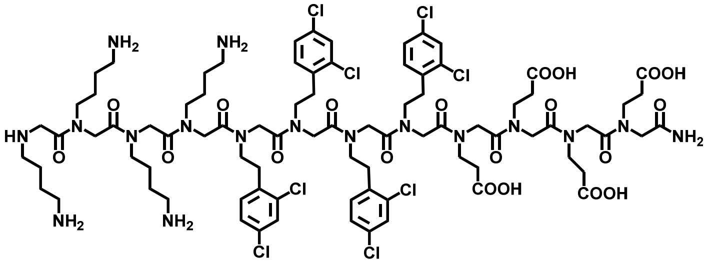

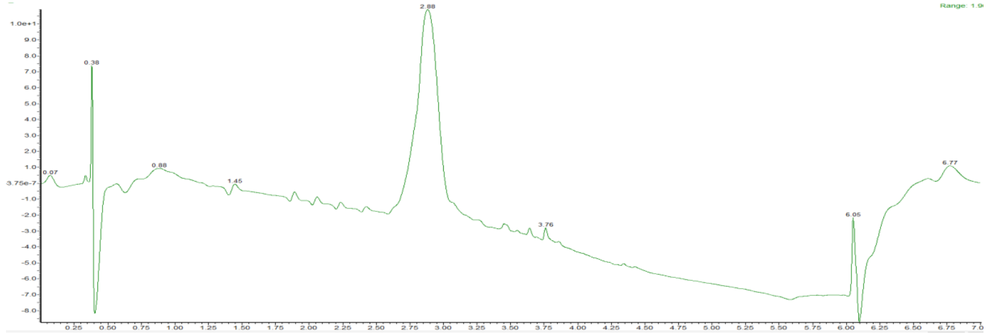

UPLC characterization of Pep-**9**

Peptoid-**10** [(Nce)$_{12}$ (Ndc)$_4$(Nba)$_4$]: 2999.5 (Molecular weight), 1000.5 (Found:[M/3+H]$^+$), 1501.3 (Found:[M/2+H]$^+$).

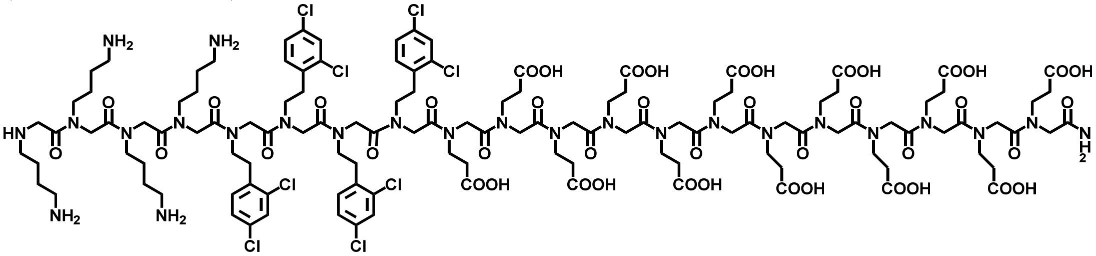

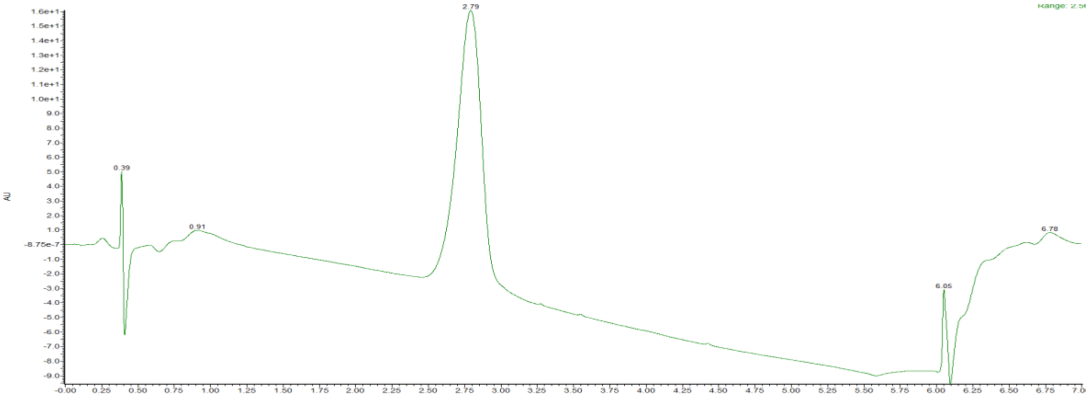

UPLC characterization of Pep-**10**

Preparation of Peptoid Stock Solution

Lyophilized peptoids (3.0×10^{-6} mol) were mixed with 1.5 mL ultrapure water in glass vial, and 10 µL saturated $(NH_4)_2CO_3$ solutions were used to facilitate dissolution. The final concentration of peptoid stock solution was 2.0 mM.

Preparation of gold nanomaterials

75 µL of 2 mM peptoid stock solution and 250 µL of 0.2 M HEPES solution were added into 175 µl of water. The mixed solution was placed for 20 mins. 6.0 µL of $HAuCl_4$ (0.1 M) was added and vortexed for 15 s, and then undisturbed at room temperature. The resulting gold nanomaterials were formed after 5 h incubation.

Preparation of Pt nanomaterials: Method 1

75 µL of 2 mM Pep-**1**, 30 µL of 0.2 M ascorbic acid (AA) solution, 250 µL of 0.2 M HEPES solution were added into 145 µL of water. 6.0 µL of K_2PtCl_4 (0.1 M) was then added, vortexed, and left undisturbed at room temperature. After 6hours, the solution were heated to 35°C. After 24 hours, Pt nanomaterials were formed.

Preparation of Pt and Pd nanomaterials: Method 2

25 µL of 2 mM Pep-**1** and 30 µL of 0.2 M AA solution were added into 445 µL of water. 6.0 µL of K_2PtCl_4 or Na_2PdCl_4 (0.1 M) was then added and vortexed. The solution was kept at 35°C. After 24 hours, Pt or Pd nanomaterials were formed.

Stability of Pt and Pd nanomaterials

The centrifuged and purified Pt or Pd nanomaterials were incubated at 60 ºC for 30 h in aqueous solution, and then used for TEM studies.

Hyperspectral UV-Vis extinction microscopy and TP-PEEM studies of plasmonic gold nanoparticles

Briefly, hyperspectral UV-Vis extinction microscopy experiments were performed using a commercial hyperspectral detector (Surface Optics 710-VP) coupled to a confocal optical microscope. In this study, the incident broad band depolarized light source was transmitted through the sample, consisting of a sparse distribution of spherical coral-shaped gold nanoparticles on a glass coverslip, using a bright field condenser. Hyperspectral extinction images are represented as $-\log(I_{sample}/I_{substrate})$, in which I_{sample} is the spatially (130 nm^2/pixel) and spectrally (375-800 nm, $\Delta\lambda = 4.7$ nm) resolved intensity across the sample, and $I_{substrate}$ is the reference hyperspectral intensity image collected from the blank coverslip.

In typical ensemble-averaged colloidal UV-Vis measurements, several factors come into play in the interpretation of the recorded optical signals. Those include but are not limited to (i) the solvent/medium of choice,(*3*) (ii) the possibility of inter-particle aggregation/coupling in

solution,(*4*) and (iii) size and structural heterogeneity.(*5*) Whereas (i) affects the recorded response in an understandable manner, both (ii) and (iii) may obfuscate the interpretation of the recorded optical spectra. In contrast, the individual particle measurements described herein exclusively report on the plasmonic response of the synthesized coral-shaped particles. However, there are limitations to this optical measurement; it is diffraction-limited, and in its present form, does not offer a direct measurement of the plasmonic enhancement factor supported by spherical coral-shaped particles. To this end, we also performed TP-PEEM measurements. The PEEM setup has been previously described elsewhere in great detail.(*6, 7*) The two aspects of multiphoton PEEM of particular interest to this report are its ability (i) to image the plasmonic fields of the synthesized plasmonic coral-shaped particles, and (ii) to provide an estimate of the enhancement factor supported by these superstructures.(*5, 8*)

Molecular dynamics simulations

To predict the conformational ensemble of the surface-adsorbed peptoids, we performed REST-MD simulations comprising a single chain of each of three peptoids, Pep-**1** (with two different protonation states, corresponding with pH 5.5 and pH 7.3), Pep-**5**, Pep-**6** and Pep-**7**, adsorbed at the aqueous Au(111) interface. To probe inter-chain interaction effects, we also carried out standard MD simulations of a two-chain surface-adsorbed peptoid system. Finally, to estimate and compare the adsorption free energy of dichlorobenzene and benzene, we performed multiple walker well-tempered metadynamics simulations, comprising a single adsorbate (dichlorobenzene and benzene) and the aqueous Au(111) interface. Simulation details are provided below.

General Simulation Set-up Details: We used an orthorhombic periodic cell, and periodic boundary conditions were applied in all three dimensions. All simulations were performed in the Canonical (NVT) ensemble, at a temperature of 300K, maintained using the Nosé-Hoover thermostat,(*9, 10*) with a coupling constant of $\tau = 0.2$ ps. Newton's equations of motion were solved using the leapfrog algorithm with an integration time-step of 1fs. Coordinates and velocities were saved every 1000 steps (1ps). Long-ranged electrostatic interactions were treated using Particle-mesh Ewald (PME),(*11*) with a cut-off at 11 Å, whereas a force-switched cut-off, starting at 9 Å and ending at 10 Å was used for Lennard-Jones non-bonded interactions.

The GolP-CHARMM(*12*) force-field was used to model the Au slab. The peptoids were described based on the force-field published by Jin and co-workers,(*2*) and water was described using the SPC/E(*13*) model. Where needed, counter-ions (Na^+ atoms) were added to ensure overall charge-neutrality of the cell, with the Dang95 interaction parameters.(*14*) All metal atoms in the slab were held fixed in space during these simulations, with only the metal atom dipoles able to freely rotate. Random initial dipole positions were used throughout. Our recent tests indicate that there is very little difference between binding obtained using a rigid substrate, vs. using a slab where all atoms can move.(*15*)

Replica Exchange with Solute Tempering Molecular Dynamics (REST-MD) Simulations: Our system comprised one peptoid chain (one of Pep-**1**, Pep-**5**, Pep-**6** or Pep-**9**); a Au slab, five atomic layers thick, presenting the (111) surface on both slab faces; and, ~11,000 SPC water molecules. The dimensions of the simulation cell were ~ 59 × 61 × 68 Å. The dimension of the

periodic cell perpendicular to the slab plane was adjusted such that the density of liquid water in the center of the space between the slab and its periodic image recovered the target density of bulk liquid water at 300 K using the SPC model.

Our implementation of REST exploits the replica exchange and free energy perturbation theory functionalities within Gromacs 5.0.1.(*16*) Details of the Terakawa implementation(*17*) of REST have been given by us previously.(*18*) In our REST simulations, we spanned an 'effective temperature' window of 300-430K with 16 replicas. The initial configurations for each replica covered a range of conformations. The adsorbate structure for each replica was initially placed such that at least one peptoid atom was found within ~3Å distance from the top surface of the Au slab. The 16 values of lambda used to scale our force-field were:
λ_j = 0.0000, 0.057, 0.114, 0.177, 0.240, 0.310, 0.382, 0.458, 0.528, 0.597, 0.692, 0.750, 0.803, 0.855, 0.930, 1.0000.

Prior to each REST simulation, initial configurations of each of the 16 replicas were equilibrated at their target potential for 0.5 ns, with no exchange moves attempted during this time. During the REST simulations, the interval between exchange attempts was set to 1000 MD steps (every 1 ps). All production REST simulations were run for a total of 20×10^6 MD steps (20 ns). Frames from the trajectory were saved every 1ps.

REST MD clustering analysis: Detailed analysis was carried out on the constant-ensemble run at an effective temperature of 300K (herein referred to as the reference trajectory). We classified the Boltzmann-weighted ensemble from our reference trajectories into groups of like structures, on the basis of similarity of their backbone structures, *via* the Daura clustering algorithm(*19*) with a root mean-squared deviation (RMSD) cutoff between the positions all peptoid backbone atoms. We used a cutoff of 2.5 Å; our extensive experience based on clustering analyses of peptides of different lengths guided our identification of this cutoff value to be reasonable in this instance. We performed our clustering analysis over the entire 20 ns trajectory in each case. The population of a given cluster was calculated as the percentage fraction of the number of frames that were assigned membership of that cluster, divided by the total number of frames in the trajectory. The cluster with the largest population corresponds with the most likely structure of the peptoid in the interface-adsorbed state.

REST MD Contact Residue analysis: To quantify residue-surface contact, first, for each reference trajectory, we calculated the distance between the topmost layer of the Au surface and each residue in the peptoid sequence. On the basis of these data, distance cut-offs were established to identify a range of separations where each particular residue was in immediate contact with the Au surface. We then calculated the fraction of frames in the reference trajectory for which each residue was found within the contact range of surface-residue separation. We then defined a residue to be a contact residue if that residue was found to bind persistently to the surface. We defined the residue-surface distance based on the vertical separation between an assigned site on each residue side-chain, and the top/bottom layer of the Au slab. These residue side-chain sites comprised the carbon atom of the carboxylate group, the nitrogen atom of the ammonium group, and the center of the aromatic ring for the Nce, Nab, and Nxpe/Ndc respectively. The cut-off distances for each residue type were set to 5.5 Å for Nce and Nab, 4.0 Å for Nxpe (*X*=H) and 4.5 Å for Ndc.

Metadynamics Simulations: The system comprised: one adsorbate (either benzene or di-chlorobenzene); a Au slab, five atomic layers thick, presenting the (111) surface on both slab faces; and, 2040 SPC water molecules. The dimensions of the simulation cell were ~ 59 × 61 × 68 Å. The dimension of the periodic cell perpendicular to the slab plane was adjusted such that the density of liquid water in the center of the space between the slab and its periodic image recovered the target density of bulk liquid water at 300 K using the SPC model.

The free energy of adsorption of both benzene and di-chlorobenzene at the aqueous Au(111) interface was calculated using GROMACS(*16*) in partnership with the PLUMED 2.2 software package.(*20*) Four different starting configurations of the adsorbate were used to perform Multiple Walkers Metadynamics simulations,(*21*) for the purpose of improving configurational sampling. A bias was applied to the centre of mass (c.o.m.) of the adsorbate in the direction perpendicular to the Au(111) surface, where the distance from the c.o.m. of the adsorbate ring to the Au(111) surface was defined as the collective variable (CV). These four initial configurations were used such that four different Gaussian potentials were added along the trajectory of the CV simultaneously at each time interval. Gaussians of 1.0 Å width were deposited every 1 ps, and the initial Gaussian height was set to 0.10 kJ mol^{-1}, while a bias factor of 10 was used.
The metadynamics simulations were run for 100 ns, in the canonical (*NVT*) ensemble at 300 K. The simulations were run until the fluctuations of the average free energy of adsorption had ceased changing appreciably as a function of time. The resulting free energy of adsorption was extracted using the integration method described in previous studies.(*22*)

Rank	Pep-1 %	Pep-5 %
**1**	45	53
**2**	17	14
**3**	10	14
**4**	5	9
**5**	5	3
**6**	4	3
**7**	3	2
**8**	2	1
**9**	1	<1
**10**	1	<1
**Total**	43	20

Supplementary Table 1. Percentage population of the top ten most populated clusters of the two surface-adsorbed peptoids, Pep-1 and Pep-5. 'Total' provides the total number of clusters, indicating the number of distinct thermally-accessible structures in each case.

	The average –Cl concentration	Cl_{2p}/Au_{4f}
Pep-**1** at pH 5.5 (1.0 mM)	3.5 AT% ± 0.17	0.10
Pep-**1** at pH 7.3 (1.0 mM)	4.0 AT% ± 0.13	0.113

Supplementary Table 2. XPS data shows that Pep-**1** exhibited higher binding affinity toward Au(111) at pH 7.3 than does Pep-**1** at pH 5.5 (AT = atomic percent).

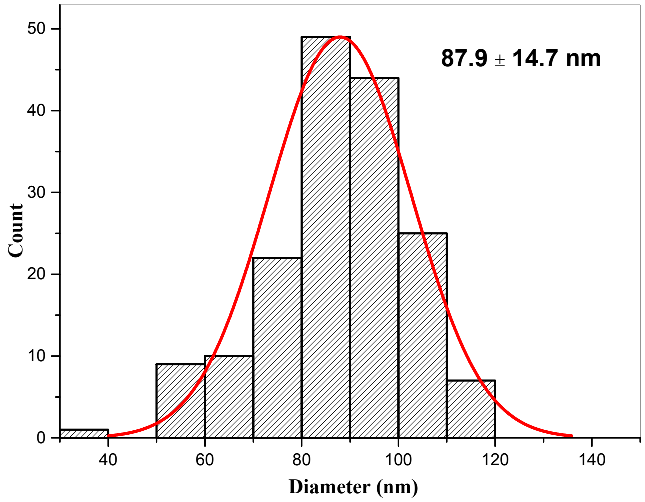

Supplementary Figure 1. Pep-1-induced spherical coral-shaped gold nanoparticles are monodisperse. The size distribution of coral-shaped particles induced by Pep-**1** [$(Nce)_8(Nab)_4(Ndc)_4$].

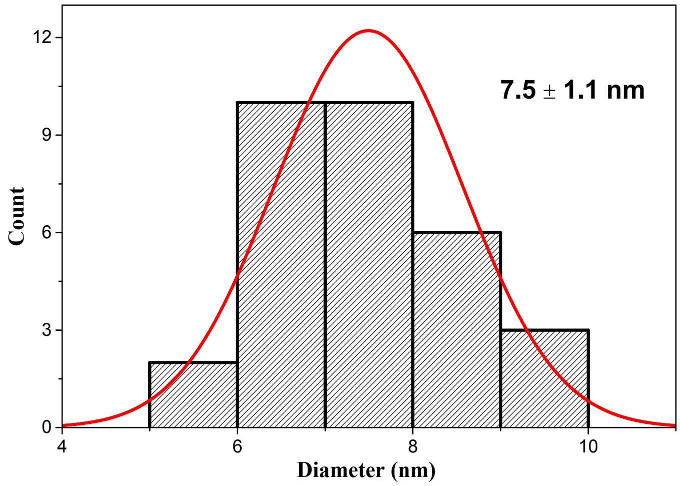

Supplementary Figure 2. The size distribution of nanorods within Pep-**1**-induced coral-shaped particles.

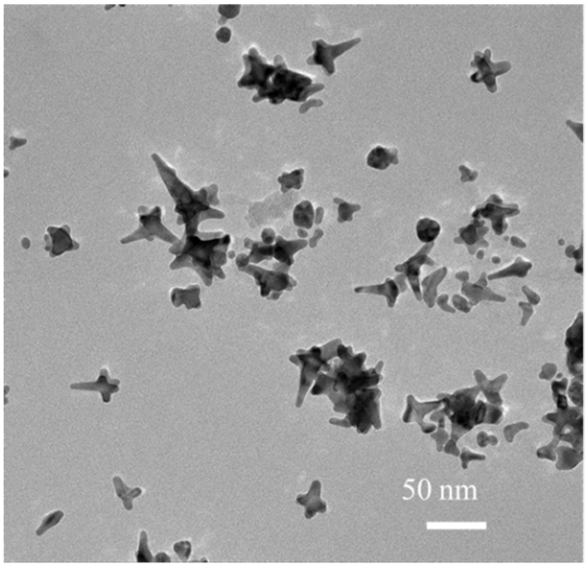

Supplementary Figure 3. TEM image showing the irregular gold nanomaterials formed in the absence of peptoids.

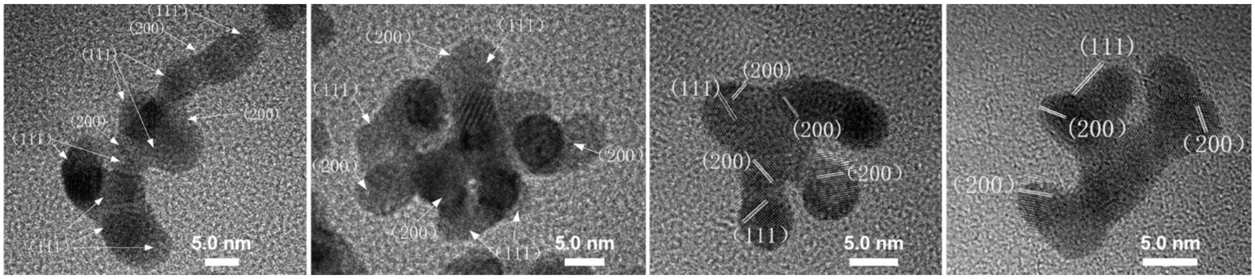

Supplementary Figure 4. High-resolution TEM of clusters of gold nanorods formed in the early stages of Pep-1-induced formation of spherical coral-shaped nanoparticles. Clusters of nanorods exhibit both (111) and (200) fringes.

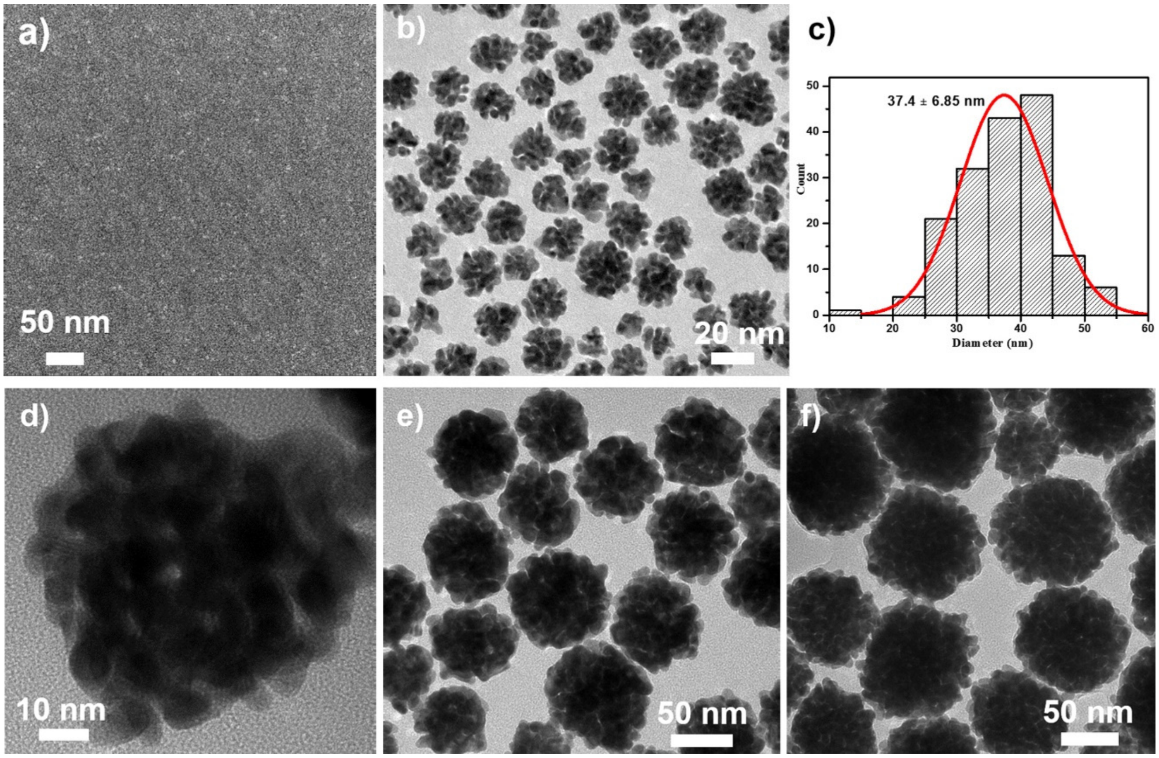

Supplementary Figure 5. **Characterizations of Pep-1-induced formation of spherical coral-shaped gold nanoparticles. a)** Negatively-stained TEM image showing that no ordered Pep-**1** assemblies were formed in the early stage of Pep-**1**-induced formation of spherical coral-shaped gold nanoparticles. 2% phosphotungstic acid was used for negative staining of the reaction solution in the early stage of Pep-**1**-induced coral-shaped particle formation. **b**) TEM image of spherical coral-shaped gold nanoparticles induced by Pep-**1** at 60 ºC and **c**) the size distribution of these particles (diameter = 37.4 nm ± 6.85 nm). **d**) TEM image showing the spherical coral-shaped gold nanoparticles induced by Pep-**1** under continuously stirring condition. **e**) TEM image showing the intact spherical coral-shaped gold nanoparticles after being incubated at 60 ºC for 30 h in aqueous solution. **f**) TEM image showing the intact spherical coral-shaped gold nanoparticles after being incubated in 1.0 M aqueous NaCl solution for 5 day.

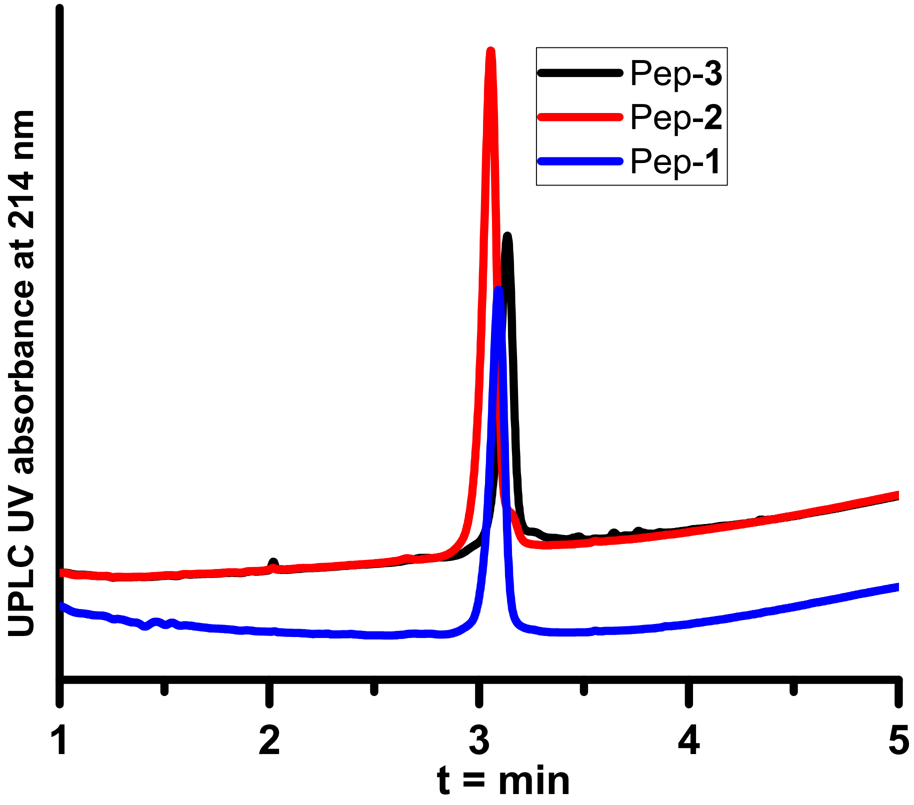

Supplementary Figure 6. Reverse phase UPLC data showing the relative hydrophobicity of Pep-1, Pep-2 or Pep-3. The longer retention time indicates higher hydrophobicity (5–95% CH_3CN in H_2O at 0.4 mL/min over 5 min; detailed UPLC conditions are provided in the peptoid synthesis section); UPLC data shows that these three peptoids exhibit minor difference in hydrophobicity.

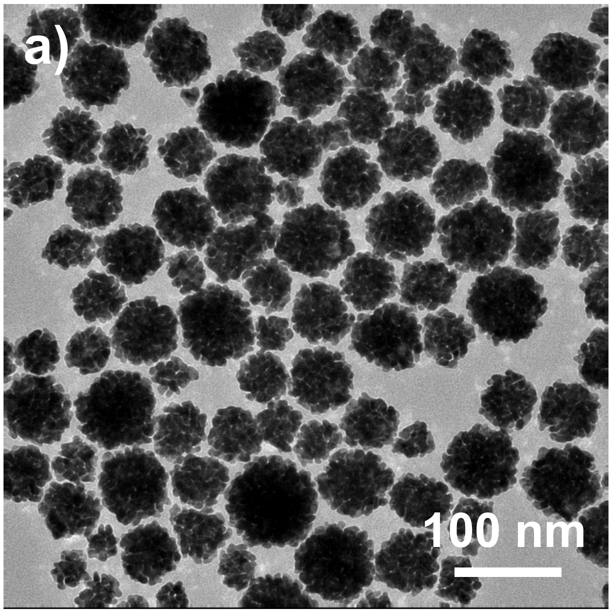
a)
100 nm

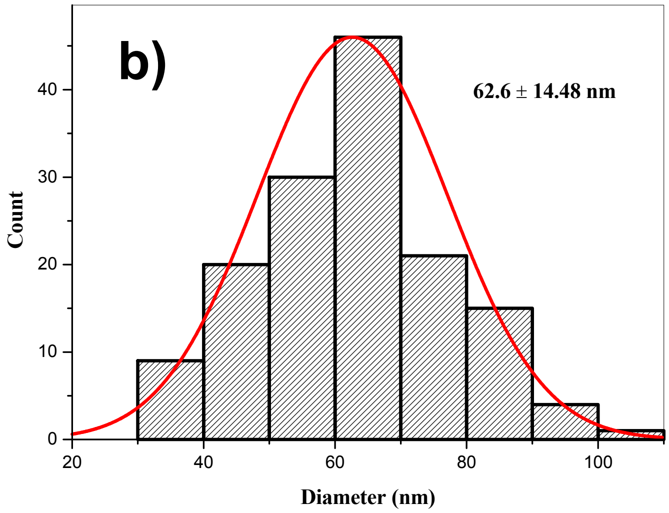
b)
62.6 ± 14.48 nm
Count
Diameter (nm)
0
10
20
30
40
20
40
60
80
100

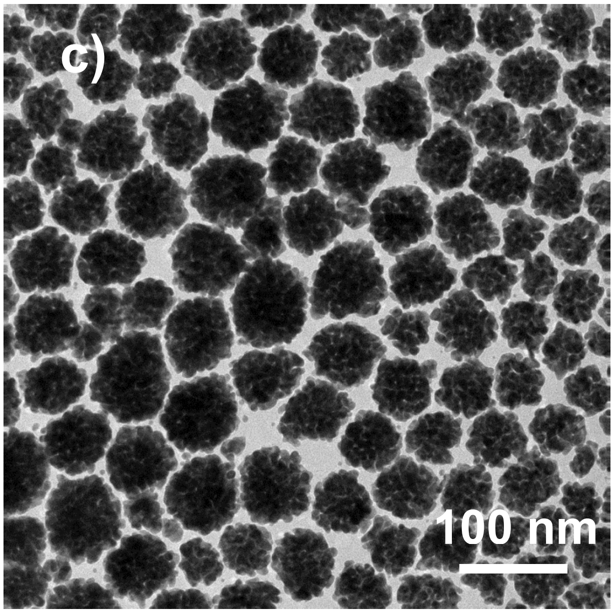
c)
100 nm

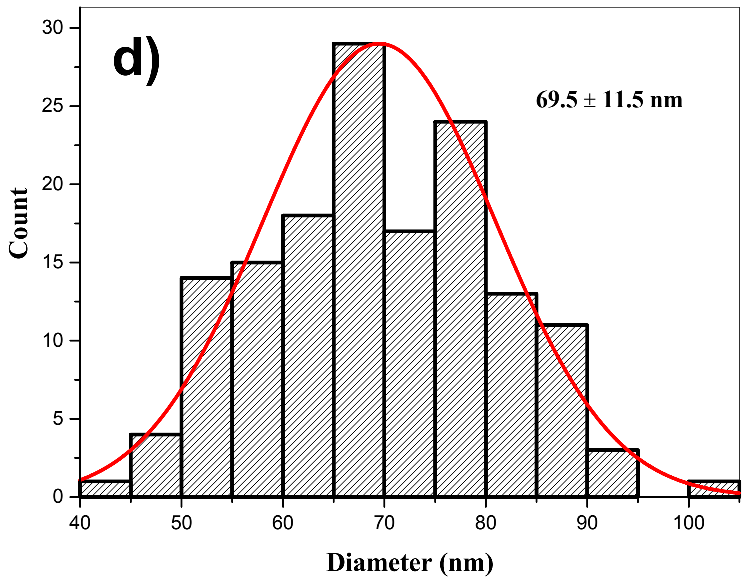

Supplementary Figure 7. Spherical coral-shaped gold nanoparticles induced by Pep-2 [$(Nce)_{12}(Nab)_4(Ndc)_4$] and Pep-3 [$(Nce)_4(Nab)_4(Ndc)_4$]. a) TEM image of spherical coral-shaped gold nanoparticles induced by Pep-**2**. **b**) The size distributions of spherical coral-shaped gold particles induced by Pep-**2**. **c)** TEM image of spherical coral-shaped gold particles induced by Pep-**3**. **d**) The size distributions of spherical coral-shaped gold particles induced by Pep-**3**.

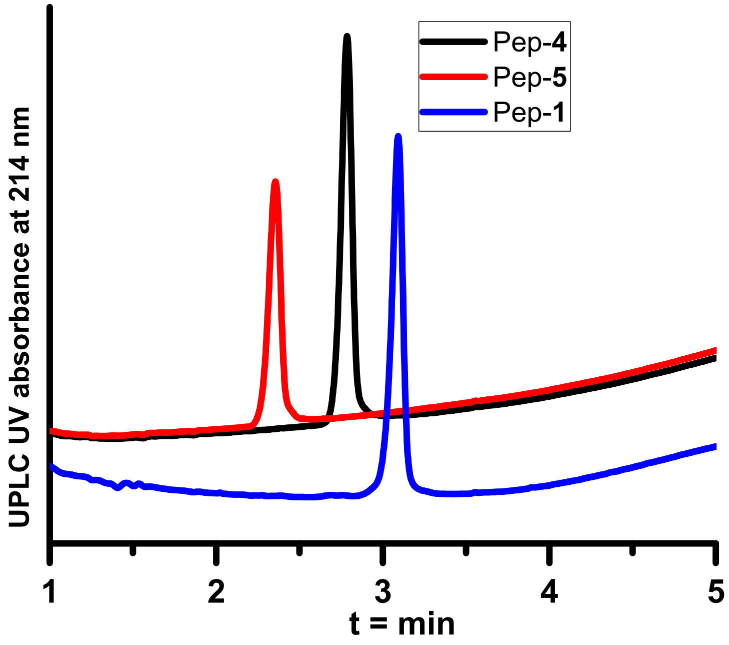

Supplementary Figure 8. Reverse phase UPLC data showing the relative hydrophobicity of Pep-1, Pep-4 or Pep-5. The longer retention time indicates higher hydrophobicity (5–95%

CH_3CN in H_2O at 0.4 mL/min over 5 min; detailed UPLC conditions are provided in the peptoid synthesis section).

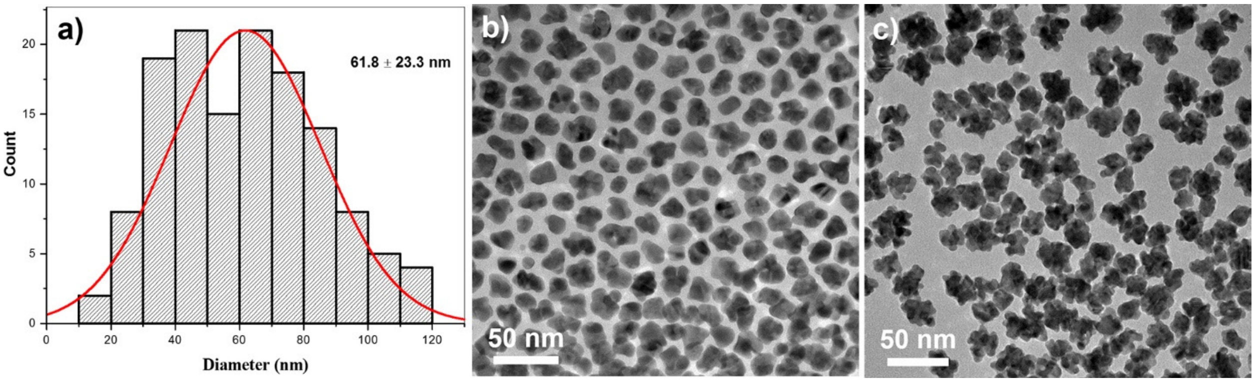

Supplementary Figure 9. Characterizations of gold nanomaterials induced by peptoids. a) The size distribution of spherical coral-shaped gold nanoparticles induced by Pep-**4**, the large standard deviation shows that the particle size is relatively polydisperse compared to those induced by Pep-**1**. **b**) TEM image of gold nanomaterials induced by Pep-**5**, these nanomaterials exhibit an irregular morphology containing almost no nanorods. **c**) TEM image of gold nanomaterials induced by Pep-**6**, the formation of these irregular particles induced by Pep-**6** suggests the importance of Nab in the formation of spherical coral-shaped gold nanoparticles.

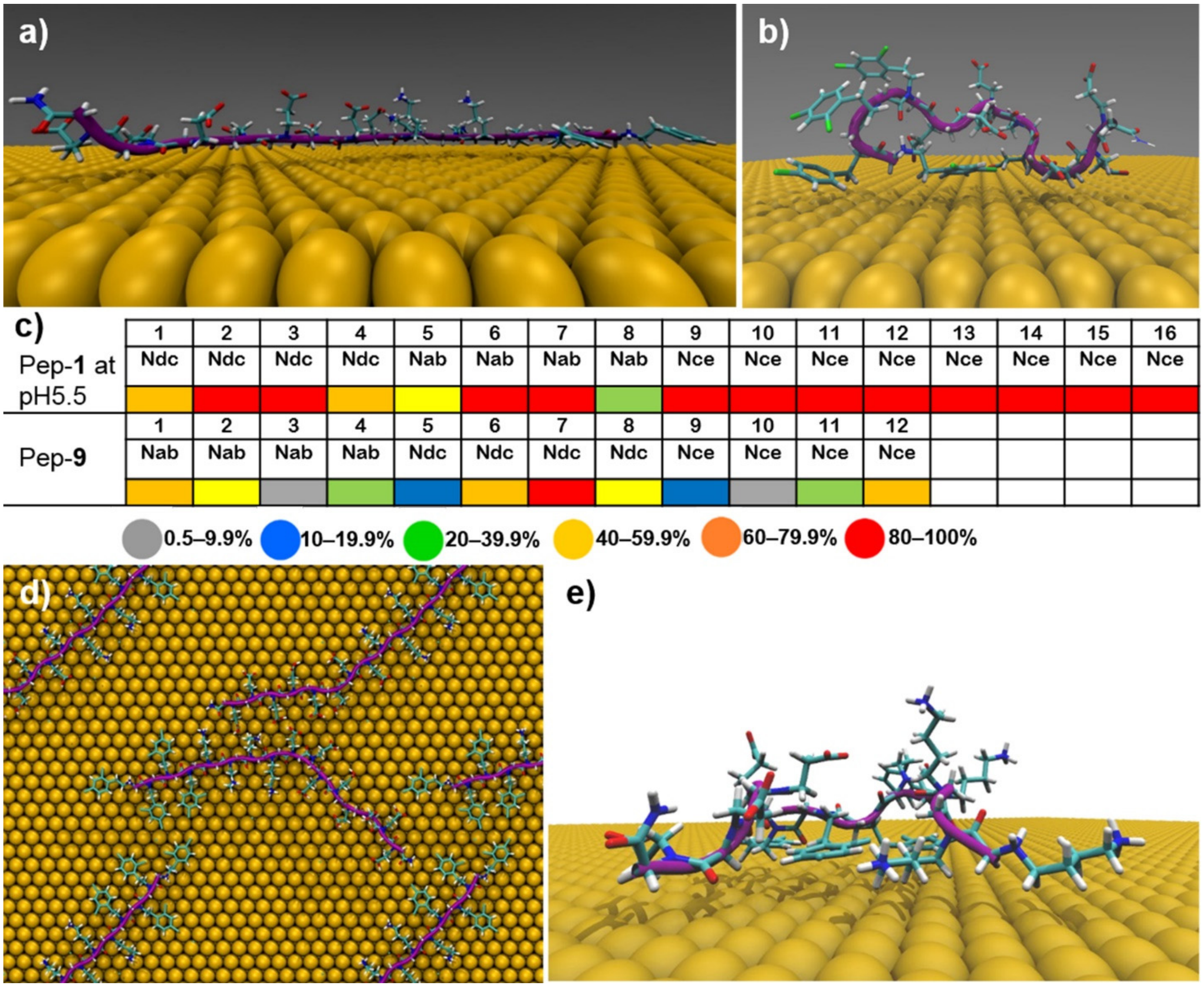

Supplementary Figure 10. Data from molecular simulations showing the binding of peptoids to Au(111) surfaces. a) A representative structure of Pep-**5** adsorbed at the aqueous Au(111) interface predicted from Replica Exchange with Solute Tempering Molecular Dynamics (REST-MD) simulations. **b**) A representative structure of Pep-**6** adsorbed at the aqueous Au(111) interface predicted from REST-MD simulations. Color code: C, cyan; O, red; N, blue; Cl, green; the peptoid backbone is colored purple. Water molecules are not shown for clarity. **c**) Average degree of peptoid-surface contact, on a residue-by-residue basis, predicted by REST-MD simulations for Pep-**1** at pH 5.5 and Pep-**9** adsorbed at the aqueous Au(111) interface. The side-chain residue numbers were enumerated from the N- to C-termini. **d**) Two-chain plan view of Pep-**1** at pH 5.5, indicating the lack of favorable inter-peptoid contacts. Color code: C, cyan; O, red; N, blue; Cl, green; the peptoid backbone is colored purple. Water molecules are not shown for clarity. **e**) A representative structure of Pep-**9** adsorbed at the aqueous Au(111) interface predicted from REST-MD simulations. Color code: C, cyan; O, red; N, blue; Cl, green; the peptoid backbone is colored purple. Water molecules are not shown for clarity.

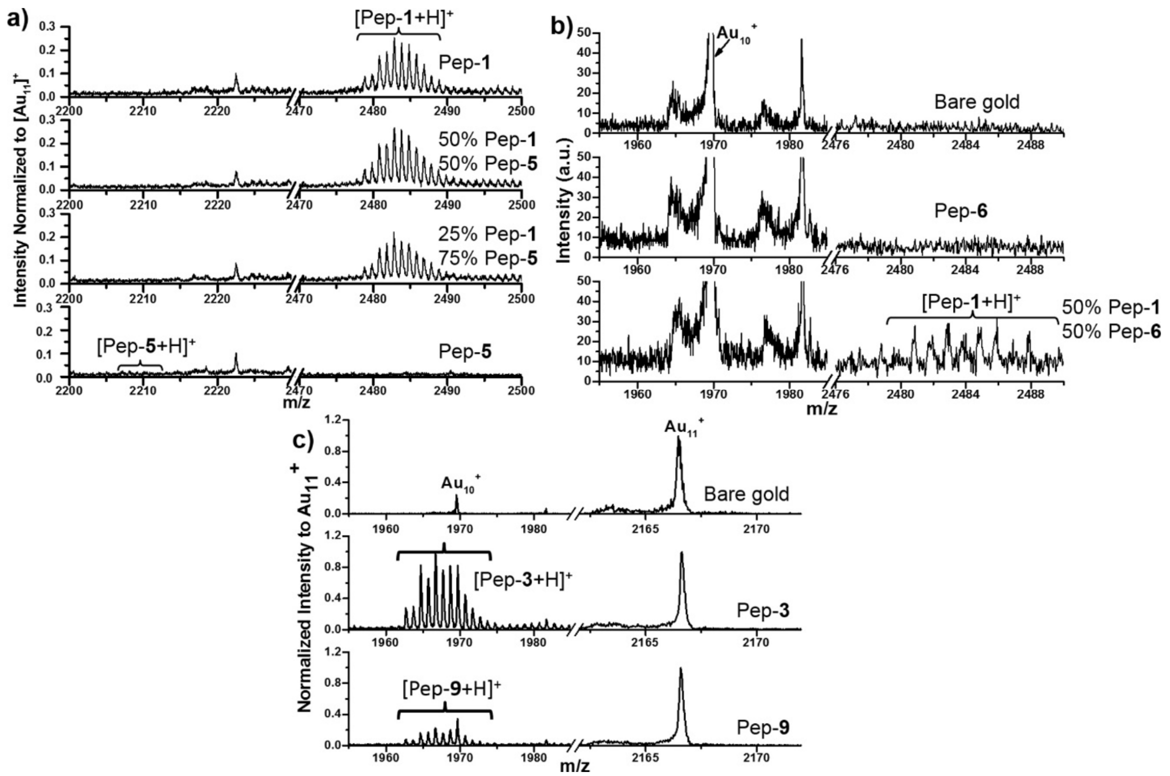

Supplementary Figure 11. ToF-SIMS data showing the strongest binding affinity of Pep-1 to Au(111) surfaces among peptoids Pep-1, Pep5 and **Pep-6, and the relatively stronger binding affinity of Pep-3 as compared to Pep-9. a**) Positive ToF-SIMS spectra on Au(111) surfaces after freshly-cleaned Au(111) surfaces were incubated with Pep-1 (1.0 mM), 50% Pep-1 (0.5 mM) mixed with 50% Pep-5 (0.5 mM), 25% Pep-**1** (0.25 mM) mixed with 75% Pep-**5** (0.75 mM) and Pep-**5** (1.0 mM). The distinct molecular ion of Pep-**1** consisting of main peaks at *m/z* = 2478.8, 2479.8, 2480.8, 2481.8, 2482.8, 2483.8, 2484.8, 2485.8, 2486.8, 2487.8, 2488.8, 2489.8 and 2490.8 was detected as long as Pep-**1** was present in the incubation solution, whereas, the molecular ion of Pep-**5** consisting of five main peaks at *m/z* = 2206.1, 2207.1, 2208.1, 2209.1, and 2210.1 were nearly undetectable no matter whether it was mixed with Pep-**1** or not, demonstrating that Pep-**1** exhibits significantly stronger adsorption onto Au(111) surfaces than Pep-**5** does. **b**) Positive ToF-SIMS spectra on Au(111) surfaces after freshly-cleaned Au(111) surfaces were incubated with H_2O(top image, bare gold), Pep-**6** (1.0 mM) (middle image) and 50% Pep-**6** (0.5 mM) mixed with 50% Pep-**1** (0.5 mM) (bottom image). In cases where Pep-**6** existed in the solution with or without Pep-**1**, its molecular ion with main peaks at *m/z* = 1966.4, 1967.4, 1968.4, 1969.4, 1970.4, 1971.4, 1972.4, 1973.4, 1973.4 and 1975.4 was not observed. It should be noted that the characteristic peak at *m/z* = 1969.7 is assigned to $[Au_{10}]^+$ ion, which appears on the spectra of all the three situations. In contrast, peaks of the molecular ion of Pep-**1** were shown after Au(111) surface was incubated with the mixture of Pep-**1** and Pep-**6**, verifying the stronger binding affinity of Pep-**1** onto Au (111) surface than that of Pep-**6**. **c**) Positive ToF-SIMS spectra on Au(111) surfaces after freshly-cleaned Au(111) surfaces were incubated with H_2O(top image, bare gold), Pep-**3** (1.0 mM) (middle image) and Pep-**9** (1.0 mM) (bottom image). Pep-**3** exhibited a significantly higher intensity of the observed molecular ion peaks at *m/z* = 1963.6, 1964.6, 1965.6, 1966.6, 1967.6, 1968.6, 1969.6, 1970.6, 1971.6 and 1972.6 compared to Pep-**9**, indicating Pep-**3** has higher binding affinity toward Au(111) than does Pep-**9**.

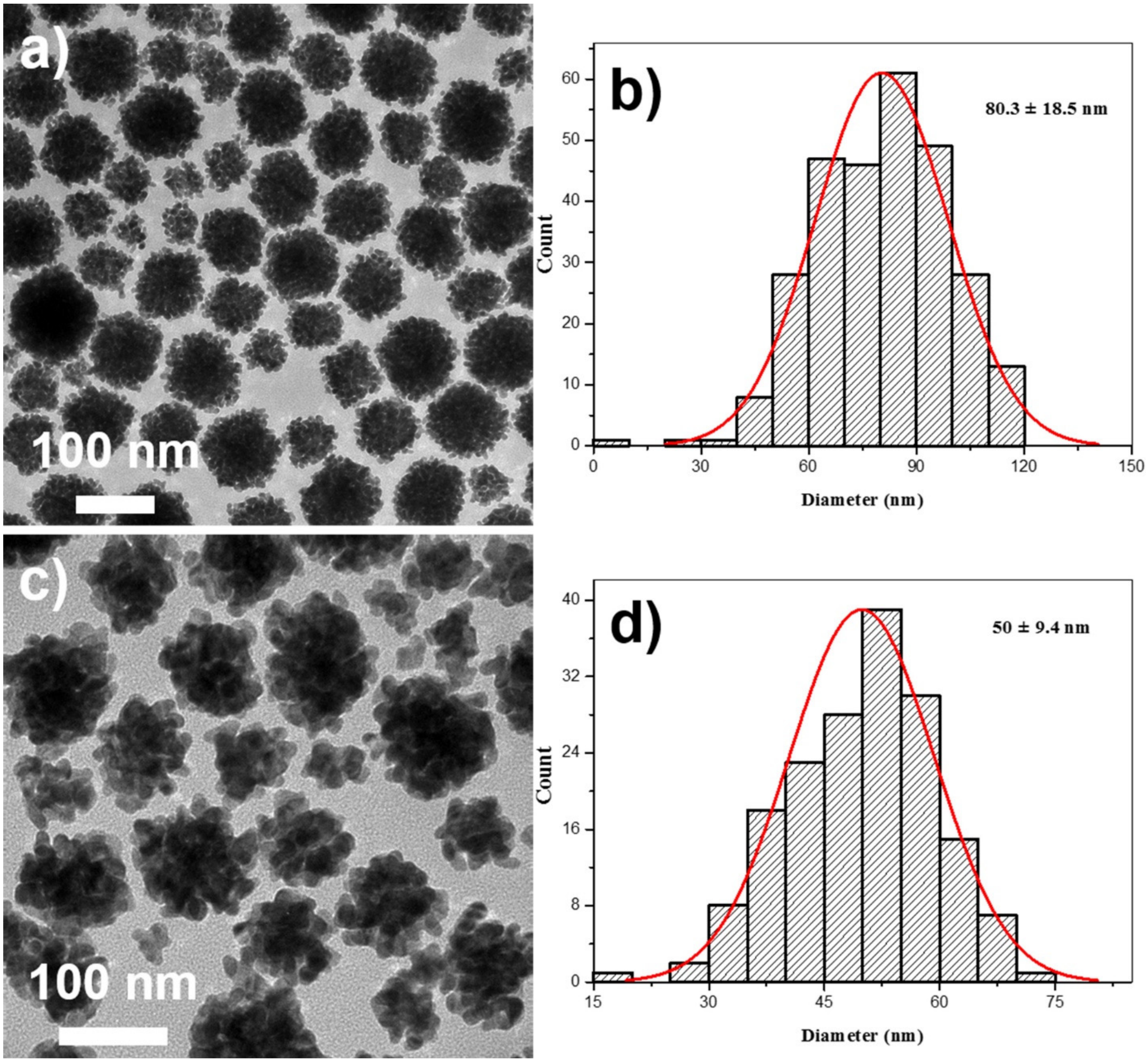

Supplementary Figure 12. Characterizations of spherical coral-shaped gold nanoparticles induced by Pep-7 and Pep-8. **a**) A TEM image showing coral-shaped gold particles induced by Pep-**7**: $(Nce)_8(Nae)_4(Nde)_4$. **b**) The size distribution of coral-shaped particles induced by Pep-**7.** **c**) A TEM image showing coral-shaped particles induced by Pep-**8:** $(Nce)_8(Nab)_1(Nde)_4$. **d**) The size distribution of coral-shaped particles induced by Pep-**8.**

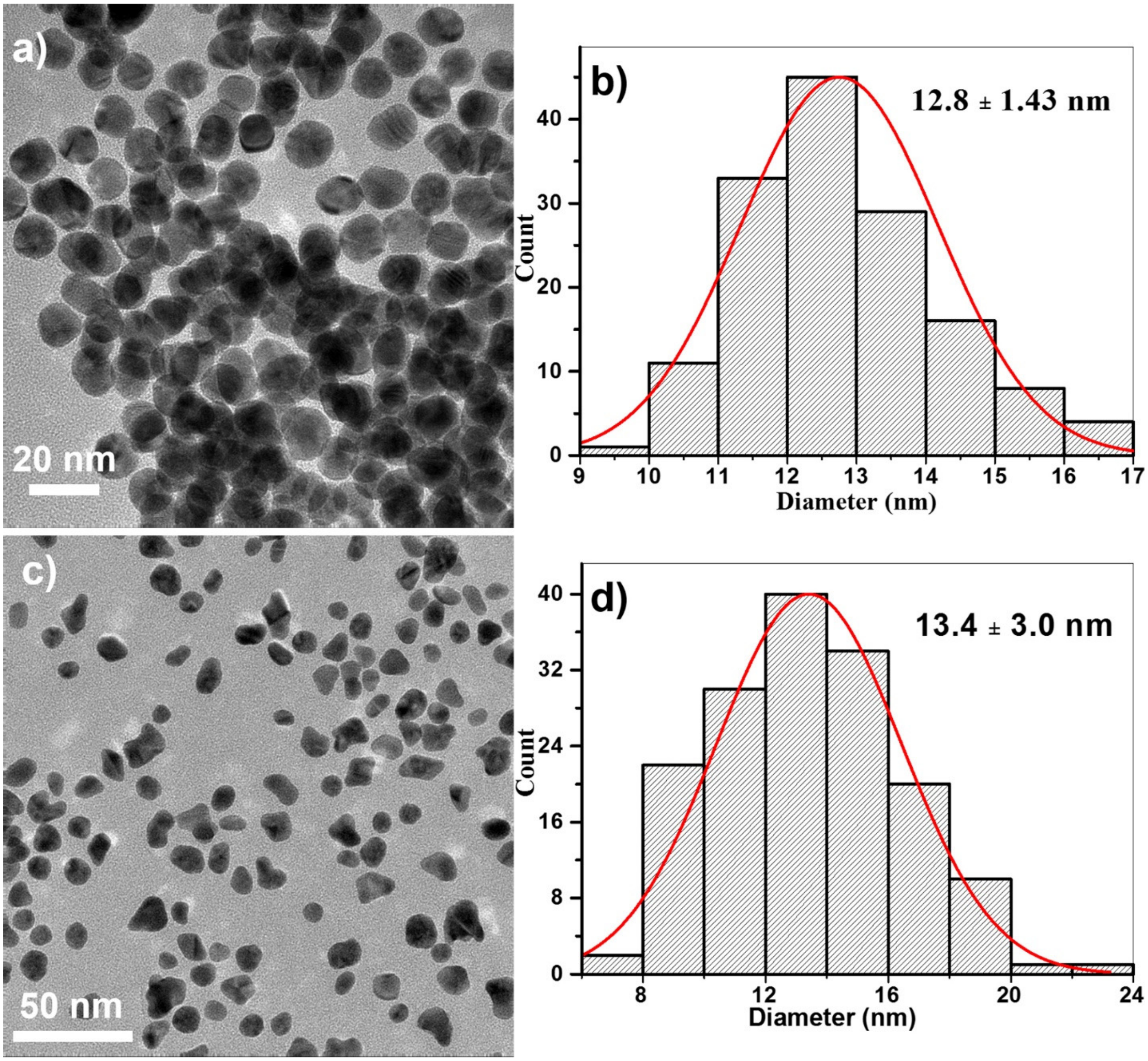

Supplementary Figure 13. Characterizations of gold nanomaterials induced by peptoids. a) A TEM image showing the nearly spherical nanoparticles induced by Pep-**9**: $(Nce)_4(Ndc)_4(Nab)_4$. **b**) The size distribution of gold nanomaterials induced by Pep-**9**. **c**) A TEM image showing the nearly spherical nanoparticles induced by Pep-**10**: $(Nce)_{12}(Ndc)_4(Nab)_4$. **d**) The size distribution of gold nanomaterials induced by Pep-**10**.

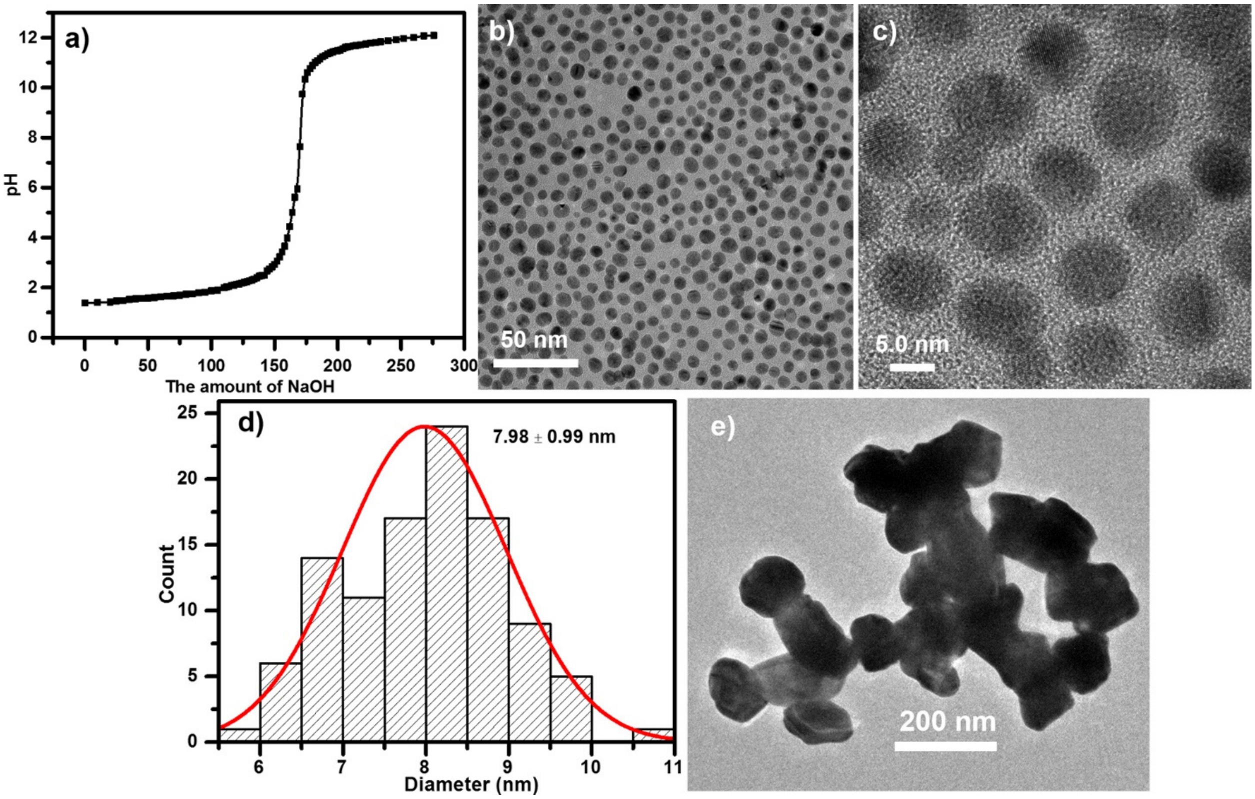

Supplementary Figure 14. Decreasing solution pH from 7.3 to 5.5 interfered with Pep-1-induced formation of spherical coral-shaped gold nanoparticles. a) Acid-base titration curve of Pep-**1**. The titration curve for Pep-1 was performed using a SevenCompct^{TM} pH/Ion meter S220. For the titration study, 0.25 mL 1.0 mM Pep-**1** aqueous solution was used. The initial pH of Pep-**1** solution was 1.38 after adding 1.0 M HCl solution. 0.1 M NaOH aqueous solution was added stepwise to adjust the pH during the titration process. **b** and **c**) TEM images of spherical gold nanoparticles induced by Pep-1 at pH5.5. **d**) The size distribution of spherical gold nanoparticles induced by Pep-**1** at pH5.5; particle size = 7.98 ± 0.99 nm (based on counting 105 different particles). **e**) The random aggregates of gold particles formed in the absence of Pep-**1** at pH5.5.

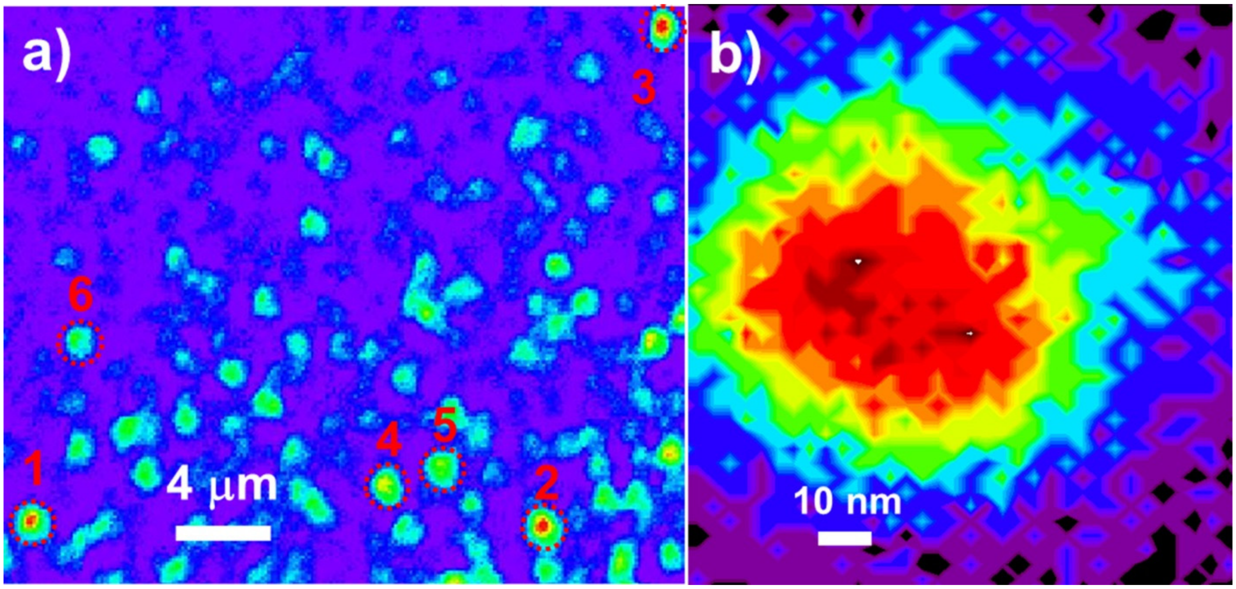

Supplementary Figure 15. Plasmonic properties of the spherical coral-shaped gold particles induced by Pep-1. a) Hyperspectral UV-Vis extinction microscopy experiments, in which six individual coral-shaped particles were identified and used for generating the hyperspectral UV-Vis extinction data described in Figure 4a. The individual coral-shaped particle absorption spectra were obtained by averaging over a total of 9 pixels (130 nm^2/pixel) centered on each particle. **b**) One more exemplary near-field enhancement map from a single isolated coral-shaped particle from high-resolution three-photon photoemission electron micrograph (TP-PEEM) imaging experiments; this data further reveals that the coral-shaped-particle center comprises the most pronounced region of plasmonic enhancement, in which the photoemission from a coral-shaped particle following p-polarized laser irradiation is normalized to the photoemission from a nominally flat gold substrate.

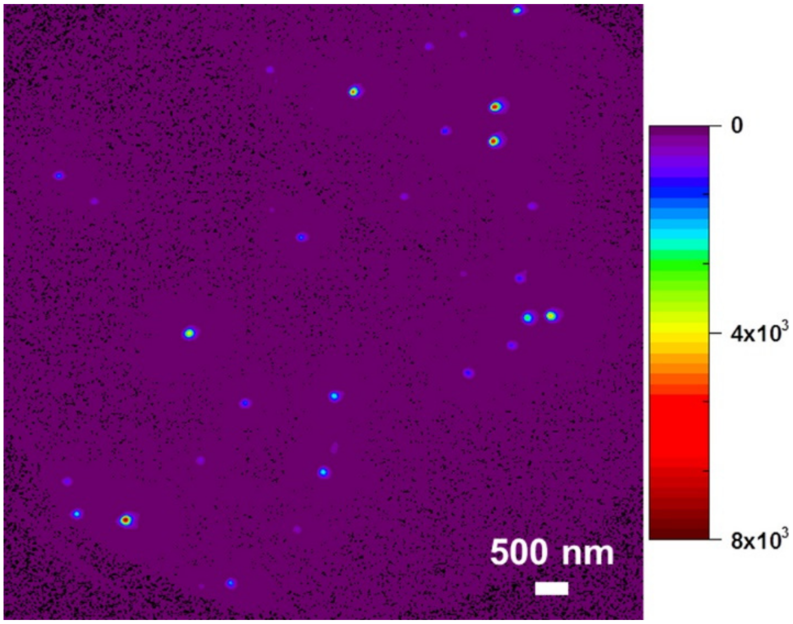

Supplementary Figure 16. Plasmonic properties of gold nanomaterials induced by Pep-5. Three-photon photoemission electron micrograph of a sparse distribution of gold nanoparticles

induced by Pep-**5** showing the photoemission enhancement map with the strongest enhancement about 10^3, which is about two orders of magnitude weaker than those generated from spherical coral-shaped gold nanoparticles induced by Pep-**1**.

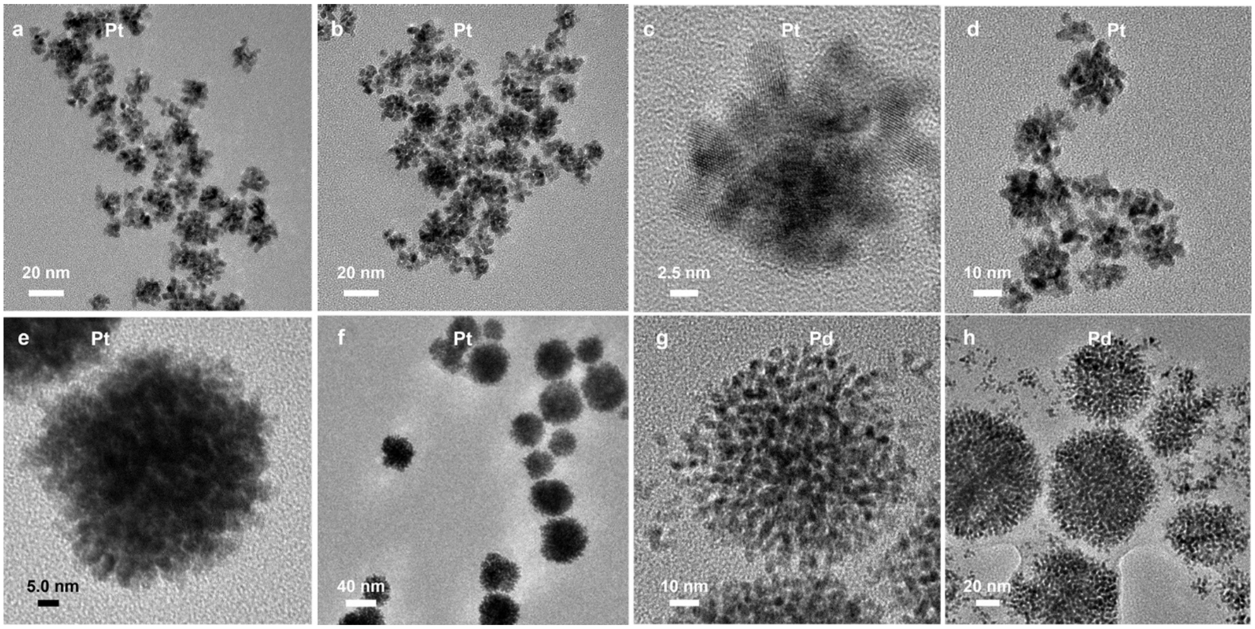

Supplementary Figure 17. Spherical coral-shaped Pt- and Pd- nanoparticles induced by Pep-1. **a** and **b**) TEM images of spherical coral-shaped Pt- nanoparticles induced by Pep-**1**. Due to the difficulty of reducing Pt^{2+} cations into Pt(0) by HEPES buffer, ascorbic acid was further added as reducing agents and the reaction was performed at 35°C to facilitate the reduction Pt^{2+} cations into Pt(0). **c** and **d**) TEM images of spherical coral-shaped Pt- nanoparticles induced by Pep-**1** in the presence of HEPES and ascorbic acid. While conditions used in the Figs. a-d is good for reducing Pt^{2+} cations into Pt(0) to form spherical coral-shaped particles, they are not suitable for reducing Pd^{2+} into Pd(0). **e** and **f**) TEM images of spherical coral-shaped Pt- nanoparticles induced by Pep-**1** using ascorbic acid only as reducing agents at 35°C. This condition is good for reducing both Pt^{2+} and Pd^{2+} to form spherical coral-shaped metallic nanoparticles. **g** and **h**) TEM images of spherical coral-shaped Pd- nanoparticles induced by Pep-**1** using ascorbic acid only as reducing agents at 35°C.

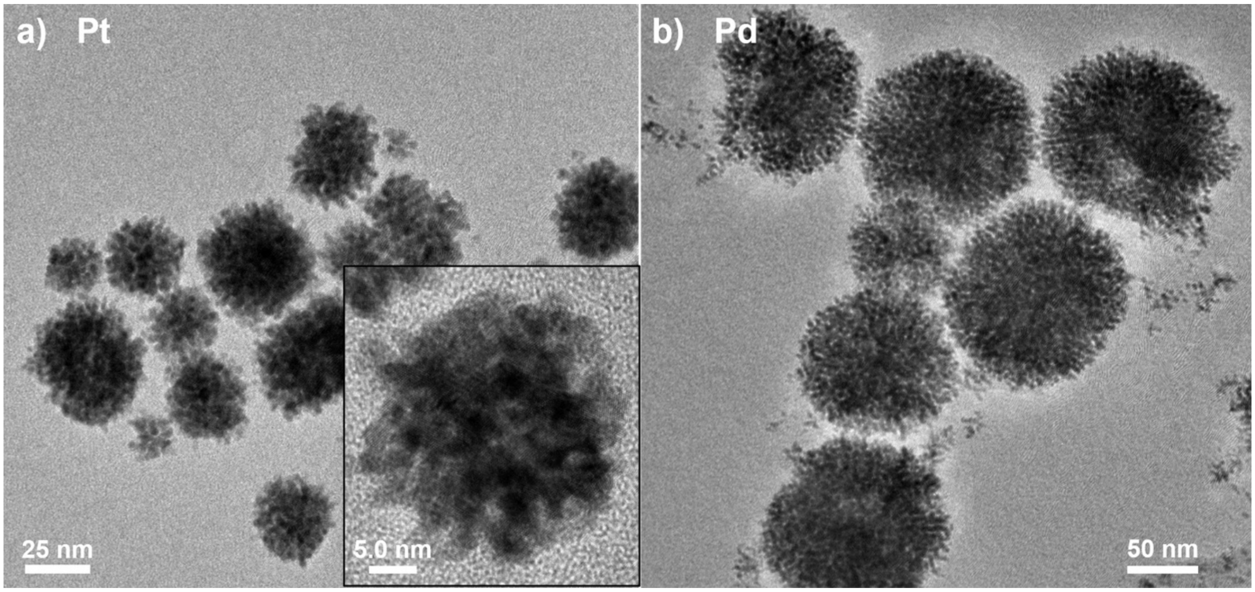

Supplementary Figure 18. The high stability of spherical coral-shaped Pt- and Pd-nanoparticles induced by Pep-1.**a**) TEM image showing that spherical coral-shaped Pt-nanoparticles are intact after being incubated at 60 ºC for 30 h in aqueous solution. The insert is a high magnification image of one spherical coral-shaped Pt- nanoparticle showing its structural similarity to those spherical coral-shaped gold nanoparticles listed in Figure 2. **b**) TEM image showing that spherical coral-shaped Pd- nanoparticles are intact after being incubated at 60 ºC for 30 h in aqueous solution.

Supplementary References:

1. R. N. Zuckermann, J. M. Kerr, S. B. H. Kent, W. H. Moos, Efficient method for the preparation of peptoids oligo(N-substituted glycines) by submonomer solid-phase synthesis. *J. Am. Chem. Soc.* **114**, 10646-10647 (1992).
2. H. Jin *et al.*, Highly stable and self-repairing membrane-mimetic 2D nanomaterials assembled from lipid-like peptoids. *Nat. Commun.*, 12252 (2016).
3. T. R. Jensen *et al.*, Nanosphere lithography: Effect of the external dielectric medium on the surface plasmon resonance spectrum of a periodic array of sliver nanoparticles. *J. Phys. Chem. B* **103**, 9846-9853 (1999).
4. D. D. Whitmore *et al.*, High Sensitivity Surface-Enhanced Raman Scattering in Solution Using Engineered Silver Nanosphere Dimers. *J. Phys. Chem. C* **115**, 15900-15907 (2011).
5. S. J. Peppernick, A. G. Joly, K. M. Beck, W. P. Hess, Plasmon-induced optical field enhancement studied by correlated scanning and photoemission electron microscopy. *J. Chem. Phys.* **138**, (2013).
6. Y. Gong, A. G. Joly, D. Hu, P. Z. E-Khoury, W. P. Hess, Ultrafast Imaging of Surface Plasmons Propagating on a Gold Surface. *Nano Lett.* **15**, 3472-3478 (2015).

7. Y. Gong, A. G. Joly, P. Z. El-Khoury, W. P. Hess, Interferometric Plasmonic Lensing with Nanohole Arrays. *J. Phys. Chem. Lett.* **5**, 4243-4248 (2014).
8. S. J. Peppernick, A. G. Joly, K. M. Beck, W. P. Hess, Plasmonic field enhancement of individual nanoparticles by correlated scanning and photoemission electron microscopy. *J. Chem. Phys.* **134**, (2011).
9. S. Nose, A Molecular-Dynamics Method for Simulations in the Canonical Ensemble. *Mol. Phys.* **52**, 255-268 (1984).
10. W. G. Hoover, Canonical dynamics: Equilibrium phase-space distributions. *Phys. Rev. A* **31**, 1695-1697 (1985).
11. T. Darden, D. York, L. Pedersen, Particle Mesh Ewald - an N.Log(N) Method for Ewald Sums in Large Systems. *J. Chem. Phys.* **98**, 10089-10092 (1993).
12. L. B. Wright, P. M. Rodger, S. Corni, T. R. Walsh, GolP-CHARMM: First-Principles Based Force Fields for the Interaction of Proteins with Au(111) and Au(100). *J. Chem. Theory Comput.* **9**, 1616-1630 (2013).
13. H. J. C. Berendsen, J. R. Grigera, T. P. Straatsma, The Missing Term in Effective Pair Potentials. *J. Phys. Chem.* **91**, 6269-6271 (1987).
14. L. X. Dang, Mechanism and Thermodynamics of Ion Selectivity in Aqueous-Solutions of 18-Crown-6 Ether - A Molecular-Dynamics Study. *J. Am. Chem. Soc.* **117**, 6954-6960 (1995).
15. L. B. Wright, C. L. Freeman, T. R. Walsh, Benzene adsorption at the aqueous (011) alpha-quartz interface: is surface flexibility important? *Mol. Simul.* **39**, 1093-1102 (2013).
16. B. Hess, C. Kutzner, D. van der Spoel, E. Lindahl, GROMACS 4: Algorithms for highly efficient, load-balanced, and scalable molecular simulation. *J. Chem. Theory Comput.* **4**, 435-447 (2008).
17. T. Terakawa, T. Kameda, S. Takada, On Easy Implementation of a Variant of the Replica Exchange with Solute Tempering in GROMACS. *J. Comput. Chem.* **32**, 1228-1234 (2011).
18. J. P. Palafox-Hernandez *et al.*, Comparative Study of Materials-Binding Peptide Interactions with Gold and Silver Surfaces and Nanostructures: A Thermodynamic Basis for Biological Selectivity of Inorganic Materials. *Chem. Mater.* **26**, 4960-4969 (2014).
19. X. Daura *et al.*, Peptide folding: When simulation meets experiment. *Angew. Chem., Int. Ed.* **38**, 236-240 (1999).
20. G. A. Tribello, M. Bonomi, D. Branduardi, C. Camilloni, G. Bussi, PLUMED 2: New feathers for an old bird. *Comput. Phys. Commun.* **185**, 604-613 (2014).
21. P. Raiteri, A. Laio, F. L. Gervasio, C. Micheletti, M. Parrinello, Efficient reconstruction of complex free energy landscapes by multiple walkers metadynamics. *J. Phys. Chem. B* **110**, 3533-3539 (2006).
22. Z. E. Hughes, T. R. Walsh, What makes a good graphene-binding peptide? Adsorption of amino acids and peptides at aqueous graphene interfaces. *J. Mater. Chem. B* **3**, 3211-3221 (2015).
