# Supplementary material for: Controlled synthesis of highly-branched plasmonic gold nanoparticles through peptoid engineering
Source: Nat Commun. 2018 Jun 13;9:2327. doi: 10.1038/s41467-018-04789-2 (PMC5998043; doi:10.1038/s41467-018-04789-2)
Supplement: Supplementary file 3 — Description of Additional Supplementary Files [file 41467_2018_4789_MOESM3_ESM.pdf]

Description of Additional Supplementary Files

File Name: Supplementary Movie 1
Description: The early stages of Pep-**1**-induced formation of spherical coral-shaped gold nanoparticles in TEM liquid cell at electron dose rate of 36.5 e/Å^2 s.

File Name: Supplementary Movie 2
Description: The early stages of Pep-**1**-induced formation of spherical coral-shaped gold nanoparticles in TEM liquid cell at electron dose rate of 36.5 e/Å^{2}s.
