# Supplementary material for: Controlled synthesis of highly-branched plasmonic gold nanoparticles through peptoid engineering
Source: Nat Commun. 2018 Jun 13;9:2327. doi: 10.1038/s41467-018-04789-2 (PMC5998043; doi:10.1038/s41467-018-04789-2)
Supplement: Supplementary file 2 — Peer Review File [file 41467_2018_4789_MOESM2_ESM.pdf]

Editorial Note: This manuscript has been previously reviewed at another journal that is not operating a transparent peer review scheme. This document only contains reviewer comments and rebuttal letters for versions considered at Nature Communications.
